# Myeloid MyD88 Mediates Macrophage Infiltration and Activation in Ang II‐Induced Cardiac Hypertrophy

**DOI:** 10.1111/jcmm.70733

**Published:** 2025-07-19

**Authors:** Ke Lin, Na Yang, Chenghong Hu, Wu Luo, Ping Huang, Guang Liang, Yi Wang

**Affiliations:** ^1^ Department of Pharmacy, Hangzhou Medical College, Zhejiang Provincial People's Hospital Affiliated People's Hospital Hangzhou Zhejiang China; ^2^ Chemical Biology Research Center, School of Pharmaceutical Sciences Wenzhou Medical University Wenzhou Zhejiang China; ^3^ School of Pharmacy Hangzhou Normal University Hangzhou Zhejiang China; ^4^ School of Pharmaceutical Sciences Hangzhou Medical College Hangzhou Zhejiang China

**Keywords:** angiotensin II, hypertrophy, inflammation, macrophages, MyD88

## Abstract

Hypertension is the leading cause of cardiac remodelling and heart failure. Recent evidence has highlighted the role of the non‐hemodynamic function of angiotensin II in hypertension. MyD88 is the canonical adaptor of TLRs and IL‐1Rs, which serves as the central node in regulating inflammatory responses. Previous studies reported the opposite roles of MyD88 in hypertension. In this study, we aimed to determine the role of MyD88 and the underlying mechanisms in hypertension. Cardiomyocyte‐specific MyD88 knockout mice, macrophage‐specific MyD88 knockout mice, and MyD88 inhibitor‐treated mice were challenged with Ang II infusion to establish a cardiac remodelling model. An inflammatory cytokine array was used to determine the internal mediators. Our results showed that cardiomyocyte MyD88 deficiency showed little protection, whereas macrophage MyD88 deficiency and pharmacological MyD88 inhibition using LM8 significantly ameliorated Ang II‐induced cardiac inflammation, fibrosis, and dysfunction. The cytokine and chemokine array demonstrated that CXCL1 and CCL2 were differentially expressed between cardiomyocyte‐specific MyD88 knockout and macrophage‐specific MyD88 knockout mice. Deletion of MyD88 in macrophages significantly decreased macrophage infiltration and suppressed the activation of macrophages, which then reduced the conditioned medium‐induced cardiomyocyte hypertrophy. Our study demonstrated that targeting myeloid MyD88 could be a potential strategy in treating hypertensive cardiac remodelling.

**Abbreviations**
Ang II: angiotensin II
CCL2: C‐C motif chemokine ligand 2
CMC‐Na: sodium carboxyl methyl cellulose
Col‐1: collagen type 1
CXCL1: C‐X‐C motif chemokine ligand 1
F4/80: EGF‐like module‐containing mucin‐like hormone receptor‐like 1
IL‐1β: interleukin 1β
IL6: interleukin 6
IκB‐α: inhibitor of NF‐κB
Lyz2: lysozyme 2
MyD88: myeloid differentiation factor 88
Myh6: myosin heavy chain 6
NF‐κB: nuclear factor‐κB p65 subunit
TGF‐β: transforming growth factor β
TNF‐α: tumour necrosis factor α
WGA: wheat germ agglutinin

## 1 Introduction

Hypertension, which affects over 1.3 billion individuals worldwide, has become the most critical and expensive public health problem [1]. Pathological cardiac remodelling secondary to hypertension has now been recognised as a critical factor for heart failure. Evidence has highlighted the role of the renin‐angiotensin system (RAS) in this process [2]. Angiotensin II (Ang II), an important mediator of RAS, is shown to be essential in hypertensive cardiac injuries because of its canonical hemodynamic properties [3]. However, lowering blood pressure using RAS blockers could not completely rescue cardiac remodelling, probably because of the non‐haemodynamic ability of Ang II, including inducing inflammation and oxidative stress [4, 5]. Therefore, we need to investigate the mechanisms mediating the non‐haemodynamic effects of Ang II in depth.

Ang II‐induced chronic inflammation is intricately linked to cardiac remodelling. It is widely accepted that hypertension is a low‐grade inflammatory disease, whereas Ang II functions as a pro‐inflammatory factor [6]. Ang II‐induced inflammatory responses in cardiomyocytes contribute to cardiac fibrosis, remodelling, and dysfunction [7]. Ang II‐induced secretion of pro‐inflammatory cytokines is believed to be an important mediator leading to cardiac remodelling. Interleukin‐targeting therapy significantly improved cardiac function and fibrosis [8, 9]. Besides, Ang II could also promote chronic inflammation in the heart via inducing chemokine secretion and recruiting infiltration of different immune cells [10]. It is reported that Ang II‐induced neutrophil accumulation occurs through the secretion of MIP‐2 and IL‐8 [11]. Wang et al. [12] also demonstrated that Ang II‐induced monocyte infiltration is mediated by the CXCL1/CXCR2 axis. CXCR2 blockade therapy also ameliorates Ang II‐induced cardiac injury [12, 13], indicating that therapies targeting inflammatory cytokines and chemokines could effectively treat Ang II‐induced cardiac hypertrophy.

MyD88 is the canonical adaptor of Toll‐like receptors (TLRs) and interleukin‐1 receptors (IL‐1Rs). MyD88 bridges external stimuli to activation of IL‐1R‐associated kinase, nuclear factor‐κB (NF‐κB), mitogen‐activated protein kinases (MAPKs), activator protein‐1 (AP‐1) and interferon regulatory factors (IRFs) [14, 15]. TLR/MyD88 signalling activation has been commonly observed in Ang II‐induced cardiac inflammation [16]. We and other groups demonstrate that inhibition of TLR signalling alleviates Ang II‐induced cardiac inflammation and fibrosis [17, 18, 19]. These studies strongly indicate that MyD88 might be a potential target for treating Ang II‐induced cardiac inflammation and fibrosis.

In this study, we explored the role of MyD88 in an Ang II‐induced mouse model of aberrant cardiac remodelling using MyD88 cardiomyocyte‐specific knockout (Myd88^f/f^ Myh6‐cre) mice, MyD88 macrophage‐specific knockout (Myd88^f/f^ Lyz2‐cre) mice, and a novel MyD88 inhibitor (LM8) [20]. We found that myeloid MyD88 contributed to the development of Ang II‐induced cardiac inflammation and fibrosis mainly by increasing the expression of CXCL1 and CCL2, which then induced the infiltration of macrophages into the heart. We further demonstrated in vitro that these infiltrated macrophages could result in cardiomyocyte hypertrophy by secreting pro‐inflammatory cytokines. Our study confirmed the role of MyD88 in Ang II‐induced cardiac remodelling and identified MyD88 as a potential target for treating hypertensive cardiac injury.

## 2 Materials and Methods

### 2.1 Reagents and Chemicals

Ang II was purchased from MedChemExpress (Monmouth Junction, NJ). LPS was obtained from Sigma‐Aldrich (St. Louis, MO). The MyD88 inhibitor LM8, which specifically binds to the TIR domain of MyD88 and inhibits its dimerisation, was previously synthesised in our laboratory [20]. The Ang II ELISA kit (#E‐EL‐H5518c) was purchased from Elabscience (Wuhan, China). Mouse brain natriuretic peptide (BNP) ELISA kit (#H166) and the creatine kinase MB isoenzyme (CK‐MB) determination kit (#H197‐1‐1) were purchased from Nanjing Jiancheng Bioengineering Institute (Nanjing, China). CFSE (#CAS:150347‐59‐4) was purchased from Santa Cruz Biotechnology (Santa Cruz, CA).

The antibodies against MyD88 (#ab2064), sarcomeric alpha actinin (#ab137346), vimentin (#ab8978), CD68 (#ab283654), IκB‐α (#ab32518), phosphorylated p65 (#ab76302), β‐Myhc (#ab50967), Col‐1 (#ab138492) and TGF‐β (#ab215715) were obtained from Abcam (Cambridge, UK). Antibodies against GAPDH were obtained from Proteintech (Wuhan, China). Antibodies against F4/80 (#sc377009) and GAPDH (#sc365062) were obtained from Santa Cruz Biotechnology. Peroxidase‐conjugated anti‐mouse (#33201ES) and anti‐rabbit (#34201ES) secondary antibodies were obtained from Yeasen Biotech (Shanghai, China).

### 2.2 Animals

All animal experiments and animal care were approved by the Animal Policy and Welfare Committee of Wenzhou Medical University (wydw2019‐0438). Animal experiments were performed following the guidelines of the National Institutes of Health (NIH, Bethesda, MD). MyD88^f/f^, Myh6‐Cre and Lyz2‐Cre mice were obtained from GemPharmatech (Nanjing, China). MyD88^f/f^ Myh6‐Cre and MyD88^f/f^ Lyz2‐Cre mice were generated from the breeding of MyD88^f/f^ with Myh6‐Cre or Lyz2‐Cre mice. C57BL/6J (Wild‐type, WT) mice were obtained from the Animal Center of Wenzhou Medical University. The genotype of the MyD88^f/f^, MyD88^f/f^ Myh6‐Cre and MyD88^f/f^ Myh6‐Cre mice, and the MyD88 knockout efficiency and specificity are shown in Figure S1. The mice were housed at constant room temperature, with a 12:12 h light–dark cycle, and fed a standard rodent diet. Mice were allowed at least 2 weeks to acclimate to the environment before starting in vivo experiments.

### 2.3 Mouse Genotyping

The genotypes of MyD88 knockout mice were identified by PCR, as previously described [21]. MasterMix (2xTaq Plus, #CW2849) and Super DNA Marker (#CW2583) were purchased from CoWin Biosciences (Beijing, China). For the MyD88^f/f^ fragment, the primers were as follows: forward primer (5′‐TGGCCCTGGTATGTAGTCTC‐3′) and reverse primer (5′‐CCTCAGTCTCACAGGTAGGTAGA‐3′), with the PCR product sizes of 419 bp (targeted) and 316 bp (wild type). For the Myh6‐Cre fragment, the mutant primers were as follows: forward primer (5′‐ATCAGAAAGGAGAATGTGGATGCTG‐3′) and reverse primer (5′‐ATGTTCACATTGGTCCAGCCACC‐3′), with a PCR product size of 602 bp (targeted). For the Lyz2‐Cre fragment, the mutant primers were as follows: forward primer (5′‐GAACACACCTGGAAGATGCTCC‐3′) and reverse primer (5′‐CATCCTTGGCACCATAGATCAGG‐3′), with a PCR product size of 718 bp (targeted). PCR conditions were as follows: denaturation at 94°C for 3 min, 94°C for 30 s, 60°C for 35 s, and 72°C for 35 s for 30 cycles, followed by extension at 72°C for 5 min. Agarose gels (2%) were used for electrophoresis. Images were obtained with a Gel Doc XR system (Bio‐Rad, Hercules, CA).

### 2.4 Development of the Ang II‐Induced Hypertensive Heart Injury Mouse Model

For the Ang II‐induced hypertensive heart injury model, mice received continuous Ang II infusion at 1 μg/kg/min, whereas control mice received equal saline via a micro‐osmotic pump for 4 weeks (Alzet MODEL 1004; San Jose, CA), as previously described [22]. The micro‐osmotic pump was implanted subcutaneously in the back of the mice. The body weight and blood pressure of the mice were recorded every 3 days.

#### 2.4.1 Model 1

12 male MyD88^f/f^ Myh6‐Cre mice and 12 male MyD88^f/f^ mice were randomly divided into four groups (*n* = 6). (1) MyD88^f/f^ group: MyD88^f/f^ mice received 4‐week infusion of normal saline; (2) MyD88^f/f^ + Ang II group: MyD88^f/f^ mice received 4‐week infusion of Ang II; (3) MyD88^f/f^ Myh6‐Cre group: MyD88^f/f^ Myh6‐Cre mice received 4‐week infusion of normal saline; (4) MyD88^f/f^ Myh6‐Cre + Ang II group: MyD88^f/f^ Myh6‐Cre mice received 4‐week infusion of Ang II.

#### 2.4.2 Model 2

10 male MyD88^f/f^ Myh6‐Cre mice and 10 male MyD88^f/f^ mice were randomly divided into four groups (*n* = 5). (1) MyD88^f/f^ group: MyD88^f/f^ mice received 4 weeks infusion of normal saline; (2) MyD88^f/f^ + Ang II group: MyD88^f/f^ mice received 4 weeks infusion of Ang II; (3) MyD88^f/f^ Lyz2‐Cre group: MyD88^f/f^ Lyz2‐Cre mice received 4 weeks infusion of normal saline; (4) MyD88^f/f^ Lyz2‐Cre + Ang II group: MyD88^f/f^ Lyz2‐Cre mice received 4 weeks infusion of Ang II.

#### 2.4.3 Model 3

24 male C57BL/6J mice were randomly divided into four groups (*n* = 6). (1) Ctrl group: C57BL/6J mice received 4 weeks infusion of normal saline; (2) Ang II group: C57BL/6J mice received 4 weeks infusion of Ang II and were intragastrically administrated with 0.5% CMC‐Na solution in the last 2 weeks; (3) Ang II + LM8‐5 group: C57BL/6J mice received 4 weeks infusion of Ang II and were intragastrically administrated with 5 mg/kg/d LM8; (4) Ang II + LM8‐10 group: C57BL/6J mice received 4 weeks infusion of Ang II and were intragastrically administrated with LM8 at the dose of 10 mg/kg/day. The dosage of LM8 used in the present study was based on our previous research [20].

After 4 weeks of modelling, the cardiac function of mice was first determined, and then the mice were sacrificed under sodium pentobarbital anaesthesia and pain medication. Heart tissues and serum were then collected for further analysis. Serum markers, including BNP and CK‐MB, were measured using commercial ELISA kits according to the manufacturer's instructions.

### 2.5 Cardiac Function Analysis

Non‐invasive transthoracic echocardiography assessed systolic cardiac function 1 day before the sacrifice. Briefly, mice were anaesthetised using isoflurane. Mouse cardiac short‐axis section indices were obtained using a Vevo‐3100 high‐resolution imaging system (Fujifilm Visual Sonics, Tokyo, Japan). Representative echocardiographs were recorded, and systolic cardiac function parameters, including ejection fraction (EF) and fractional shortening (FS), were analysed.

### 2.6 Tissue Staining and Histology Analysis

The fixed heart tissues were embedded in paraffin and were cut into 5‐μm sections. The heart sections were first dehydrated and stained with a haematoxylin and eosin (H&E) kit following the manufacturer's protocol. For the analysis of collagen deposition, Masson's staining and Sirius red staining were performed using commercial kits according to the manufacturer's protocol. Representative histology images were recorded using a light microscope (Nikon, Tokyo, Japan).

For immunochemistry staining, paraffin‐embedding heart sections were deparaffinised, rehydrated and then subjected to antigen retrieval in 0.01 mol/L citrate buffer (pH 6.0) for 3 min at 98°C. Heart sections were then blocked with 5% BSA for 1 h, and incubated with the primary antibody against F4/80 (1:200) overnight at 4°C, followed by incubation with the anti‐mouse secondary antibodies (1:200). The reaction was visualised with DAB solution. Sections were then counterstained with haematoxylin and viewed and captured under the Nikon light microscope (Nikon).

For immunofluorescence staining, frozen heart sections (5‐μm thick) were fixed and permeabilised with 0.1% Triton, followed by blockage with 5% BSA. Sections were then incubated with the primary antibody (anti‐MyD88, 1:200; anti‐α‐actinin, 1:200; anti‐Vimentin, 1:200; anti‐CD68, 1:200; anti‐WGA‐FITC) overnight at 4°C, followed by incubation with the secondary antibodies (anti‐mouse 488 and anti‐rabbit TRITC, 1:200). Sections were then counterstained with DAPI and viewed and captured using a Leica TCS SP8 confocal laser scanning microscope (Leica, Solms, Germany).

### 2.7 Cell Culture

Immortalised rat cardiomyocyte cell line H9c2 was purchased from the Shanghai Institute of Biochemistry and Cell Biology (Shanghai, China). H9c2 cells were cultured in Dulbecco's Modified Eagle's Medium (DMEM), supplemented with 10% fetal bovine serum, 100 U/mL penicillin, and 100 U/mL streptomycin.

Mouse peritoneal macrophages (MPMs) were isolated as previously described [23]. Peritoneal macrophages were collected 2 days after intraperitoneal injection of thioglycolate solution. The cell suspension was seeded and cultured with RPMI‐1640 medium supplemented with 10% FBS, 100 U/mL penicillin, and 100 mg/mL streptomycin.

Neonatal murine ventricular myocytes (NMCMs) were isolated as previously described [24]. In brief, hearts from 1‐ or 2‐day‐old neonatal mice were harvested and digested with collagenase II (Biosharp, Shanghai, China) and trypsin (Solarbio, Beijing, China). After the differential adhesion to remove the fibroblasts, the remaining cell suspension was then seeded and cultured with DMEM supplemented with 10% FBS, 100 U/mL penicillin, and 100 mg/mL streptomycin.

For in vitro experiments, MPMs were challenged with 1 μM Ang II. For the adhesion experiment, isolated MPMs were first labelled with CFSE in tubes for 30 min, then challenged with Ang II for 6 h, and then seeded into plates that were culturing H9c2 cells. After 4 h, the medium was removed and the plate was washed with PBS 3 times and then viewed and captured using a Nikon fluorescence microscope (Nikon).

### 2.8 Conditioned Medium and Rhodamine Phalloidin Staining

The conditioned medium was used to explore the effects of macrophages on cardiomyocytes, as described previously [25]. Briefly, MPMs isolated from MyD88^f/f^ Lyz2‐Cre and MyD88^f/f^ mice were treated with 0.5 μg/mL LPS or PBS for 24 h, and the medium was collected as the conditioned medium. H9c2 cells were then challenged with the conditioned medium for 24 h by adding the conditioned medium into the cultured medium at a ratio of 1:1.

For Rhodamine‐phalloidin staining, cells were fixed with 4% paraformaldehyde, permeabilised with 0.1% Triton X, and then stained with 50 ng/mL rhodamine phalloidin for 30 min at room temperature. Slides were then counterstained with DAPI for 10 min. Immunofluorescence was viewed and captured using a Nikon epifluorescence microscope.

### 2.9 Real‐Time Quantitative PCR

Total mRNA was extracted from cells or heart tissues using TRIZOL (Thermo Fisher; Waltham, MA). The two‐step PrimeScript reverse transcription reagent Kit (Perfect Real Time; TAKARA) and the Mastercycler ep realplex detection system (Eppendorf, Hamburg, Germany) were used to perform reverse transcription and quantitative PCR assay. Primers for genes from Thermo Fisher are shown in Table S1. The relative amount of all genes was normalised to the amount of β‐actin.

For the inflammatory cytokine and chemokine array assay, mouse cDNAs were mixed in each group and used as a single sample in the analysis. After the exclusion of genes with abnormal melting curves, the data were normalised to β‐actin and then compared to the MyD88^f/f^ group. The heat map was used to represent the data using online tools (https://www.omicstudio.cn).

### 2.10 Western Blotting Assay

Heart tissues were homogenised and centrifuged to collect total proteins. Lysates were separated by 10% SDS‐PAGE and transferred to PVDF membranes. Membranes were then blocked in Tris‐buffered saline containing 0.05% Tween 20 and 5% non‐fat milk for 1.5 h. After washing with TBST, the membranes were incubated with the primary antibodies and subsequent secondary antibodies conjugated to horseradish peroxidase. Immunoreactive bands were detected using the chemi‐luminescence reagent (Bio‐Rad) and visualised with the ChemiDoc XRS+ system (Bio‐Rad, Carlsbad, California, USA). GAPDH was used as the loading control. ImageJ analysis software (NIH) was used for densitometric quantification of blots.

### 2.11 Statistical Analysis

Statistical analysis was performed using GraphPad Prism 8.0 (San Diego, CA). Data shown were from three independent experiments and were expressed as mean ± SEM. Student's *t*‐test was applied when comparing two data groups. One‐way ANOVA followed by Dunnett's post hoc test was used when comparing more than two data groups. *p* < 0.05 was considered significant in statistics. Post‐tests were run if *F* achieved *p* < 0.05 and there was no significant variance in homogeneity.

## 3 Results

### 3.1 Ang II Elevates the MyD88 Expression in Heart Tissue

**FIGURE 1:**
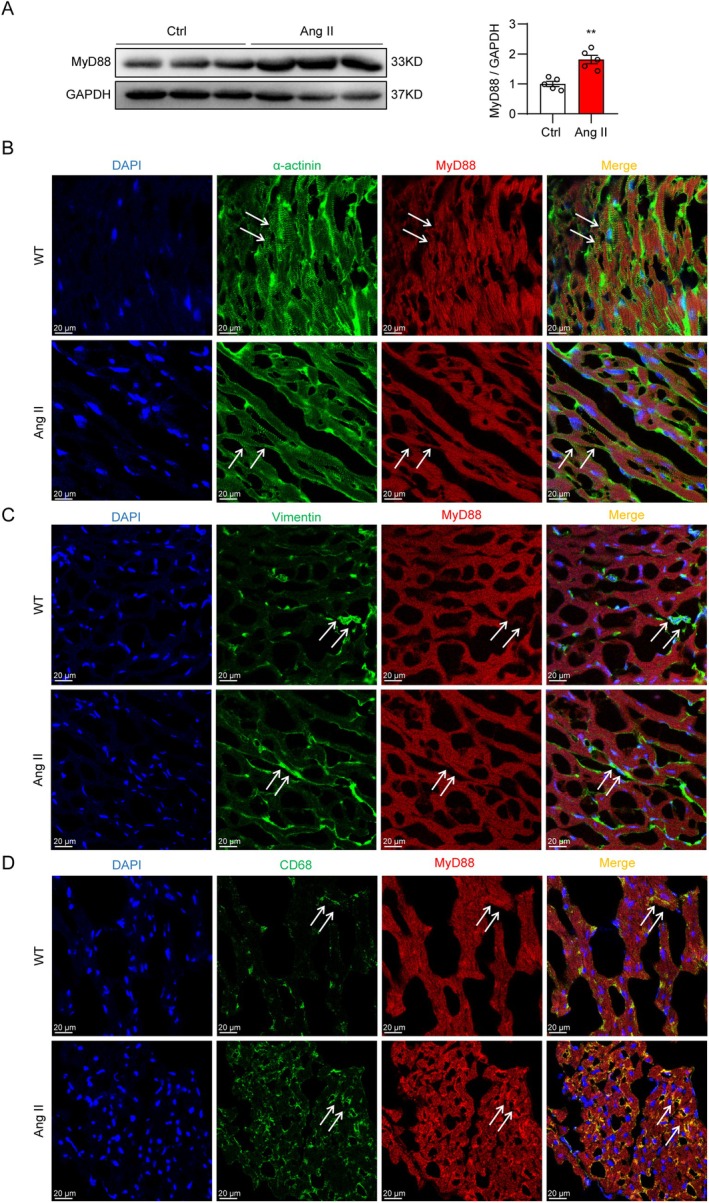
Ang II treatment increases the expression of MyD88 in mouse heart tissues. C57BL/6 mice were continuously challenged with Ang II (1 μg/kg/min) or normal saline using micro‐osmotic pumps for 4 weeks. (A) Protein levels of MyD88 in heart tissues from normal (Ctrl) and Ang II‐treated (Ang II) mice were measured using Western blot analysis. GAPDH was used as the loading control. Representative Western blot images (left panel) and densitometric quantifications (right panel) are shown. (B–D) The cellular distribution of MyD88 in heart tissues from normal and Ang II‐infused mice was determined using the immunofluorescence assay. Representative immunofluorescence images of MyD88 (red) in α‐actinin staining‐positive cardiomyocytes (green), vimentin staining‐positive fibroblasts (green), and CD68 staining‐positive macrophages (green) are shown. Sections were counterstained with DAPI (blue). Scale bar = 20 μm. Statistical data were presented as mean ± SEM, *n* = 5; **p < 0.01 compared to the control group (Ctrl).

To explore the role of MyD88 in Ang II‐induced cardiac remodelling, we first evaluated the expression of MyD88 in normal and hypertensive heart tissues. We found that Ang II caused significantly elevated MyD88 expression in heart tissue (Figure 1A). Immunofluorescence staining of MyD88 was then performed to determine the cellular source of the protein. We confirmed that MyD88 immunoreactivity was lacking in vimentin‐positive fibroblasts but mainly presented in α‐actinin‐positive cardiomyocytes and CD68‐positive macrophages (Figure 1B–D). In addition, the co‐location of CD68 and MyD88 revealed an increased infiltration of macrophages and expression of MyD88 upon Ang II infusion (Figure 1D).

### 3.2 Cardiomyocyte Deletion of MyD88 Shows Few Improvements in Ang II‐Induced Cardiac Hypertrophy

Since MyD88 was mainly expressed in cardiomyocytes and macrophages, we first explored the role of cardiomyocyte MyD88 in Ang II‐induced cardiac remodelling. We crossed MyD88^f/f^ mice with the cardiomyocyte‐specific Cre mice (Myh6‐Cre) to obtain MyD88^f/f^ Myh6‐Cre mice, which selectively knocked out MyD88 in cardiomyocytes. MyD88^f/f^ Myh6‐Cre mice and MyD88^f/f^ littermates were treated with continuous Ang II infusion through a micro‐osmotic pump for 4 weeks. No changes were observed in body weight among the 4 groups (Figure S2A). Also, MyD88 cardiomyocyte‐specific knockout did not reverse the Ang II infusion‐induced elevated systolic blood pressure when compared to the MyD88^f/f^ mice (Figure S2B). Besides, no significant changes in serum Ang II levels were observed between MyD88^f/f^ Myh6‐Cre mice and MyD88^f/f^ mice treated with Ang II (Figure S2C). All these data indicated that cardiomyocyte deletion of MyD88 did not affect the hypertension and serum Ang II level.

Myocardial remodelling results in heart dysfunction. Therefore, we performed non‐invasive echocardiography. As shown in Table 1 and Figure S2E, mice from the MyD88^f/f^ + Ang II group showed significantly lower ejection fraction (EF%) and fractional shortening (FS%). However, cardiomyocyte deletion of MyD88 showed few protections, as no significant changes in EF and FS were observed in MyD88^f/f^ Myh6‐Cre + Ang II mice compared to MyD88^f/f^ + Ang II mice (Table 1, Figure S2E). In addition, heart weight to body weight ratio (HW/BW), heart weight/tibial length (HW/TL), and serum CK‐MB also indicated the point (Table 1). These data indicated that cardiomyocyte‐specific knockout of MyD88 showed no significant protective effect against Ang II‐induced cardiac dysfunction in mice.

**TABLE 1:** Biometric and echocardiographic parameters of the Model 1 experimental mice.

Parameters	MyD88^f/f^ (*n* = 6)	MyD88^f/f^ (*n* = 6)	MyD88^f/f^ MyH6‐Cre (*n* = 6)	MyD88^f/f^ MyH6‐Cre (*n* = 6)
		Ang II (pump)		Ang II (pump)
Heart rate, bpm	433.21 ± 9.57	435.75 ± 15.04	467.43 ± 9.61	446.43 ± 12.71
EF, %	60.99 ± 1.35	54.28 ± 2.15*	61.43 ± 1.65	54.54 ± 0.98^ns^
FS, %	32.14 ± 0.96	27.7 ± 1.34*	32.59 ± 1.14	27.71 ± 0.69^ns^
LVAWs, mm	1.36 ± 0.04	1.37 ± 0.04	1.35 ± 0.04	1.42 ± 0.08
LVAWd, mm	0.92 ± 0.03	0.99 ± 0.01	0.88 ± 0.02	1.02 ± 0.04
LVPWs, mm	1.19 ± 0.03	1.25 ± 0.09	1.19 ± 0.03	1.37 ± 0.08
LVPWd, mm	0.86 ± 0.03	0.79 ± 0.04	0.89 ± 0.03	0.98 ± 0.06
Weight, g	29.3 ± 0.46	28.24 ± 0.53	28.82 ± 0.57	29.37 ± 0.98
HW/BW, mg/g	5.19 ± 0.09	5.95 ± 0.33*	5.24 ± 0.11	5.85 ± 0.21^ns^
HW/TL, mg/cm	8.78 ± 0.04	9.93 ± 0.59*	8.46 ± 0.17	9.89 ± 0.33^ns^
CK‐MB, U/L	172.42 ± 32.58	291.83 ± 23.78*	149.31 ± 19.01	223.87 ± 21.79^ns^

*Note:* *p < 0.05, compared to MyD88^f/f^ group; ^ns^ *p* > 0.05, compared to MyD88^f/f^ + Ang II group.

**FIGURE 2:**
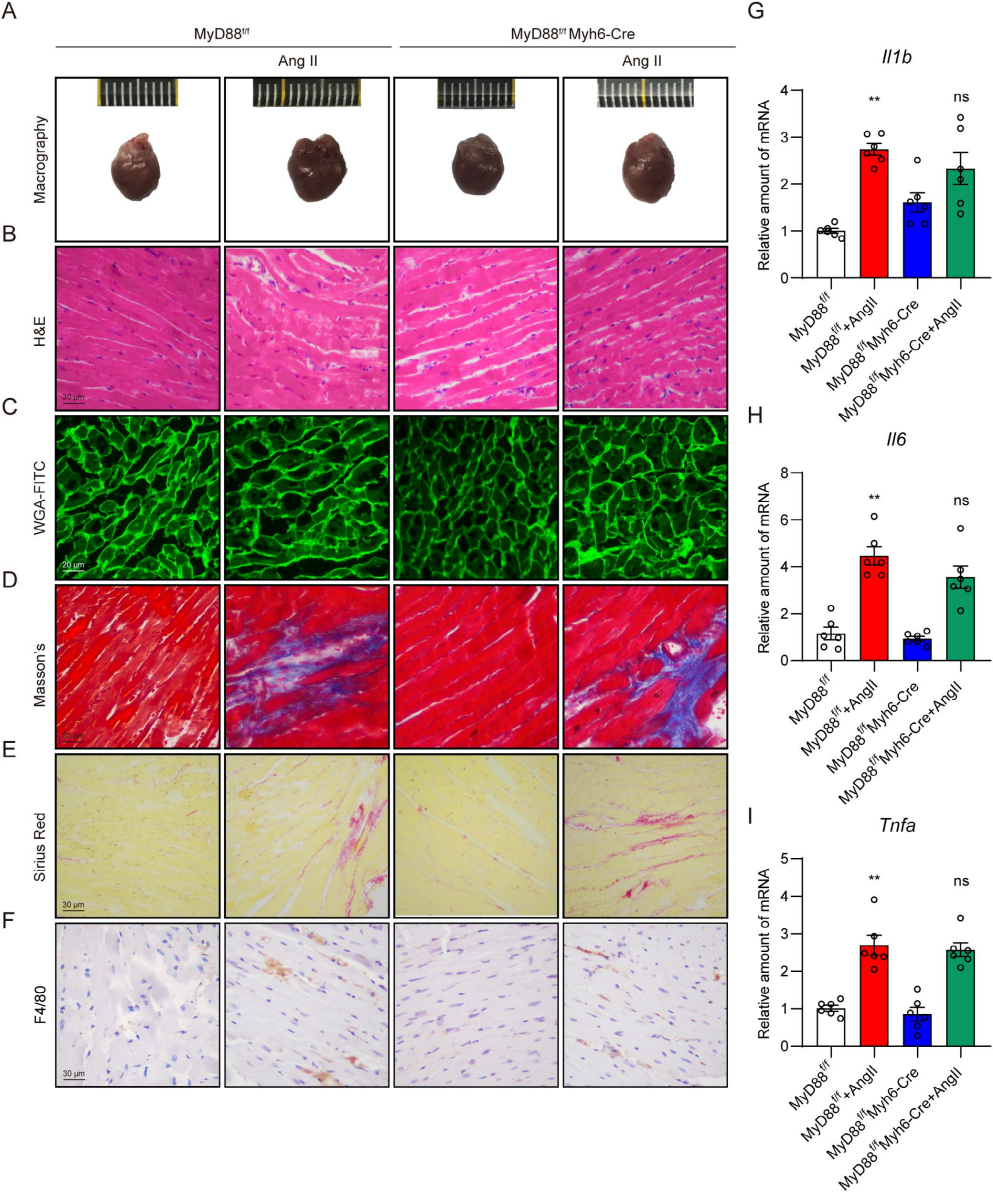
Cardiac‐specific knockout of MyD88 exhibits no significant therapeutic effect on Ang II‐induced cardiac hypertrophy. MyD88^f/f^ and MyD88^f/f^ Myh6‐Cre mice were continuously challenged with Ang II (1 μg/kg/min) or normal saline using micro‐osmotic pumps for 4 weeks. (A) Representative images of heart tissues. (B) Haematoxylin & Eosin staining showing myocardial fibre arrangements in heart tissues [scale bar = 30 μm]. (C) WGA‐FITC staining showing the cross‐sectional area of cardiomyocytes in heart tissues [scale bar = 20 μm]. (D) Masson's staining showing the interstitial fibrotic area in heart tissues [scale bar = 30 μm]. (E) Sirius Red staining showing the heart tissue fibres [scale bar = 30 μm]. (F) Anti‐F4/80 staining showing macrophage infiltration in heart tissues [scale bar = 30 μm]. (G–I) RT‐qPCR analysis of *Il1b* (G), *Il6* (H) and *Tnfa* (I) in heart tissues. Data were presented as mean ± SEM, *n* = 6; **p < 0.01 compared to the MyD88^f/f^ group; ns = not significant, compared to the MyD88^f/f^ + Ang II group.

Cardiac fibrosis is a hallmark of pathological remodelling in Ang II‐induced cardiac dysfunction. We then detected cardiac fibrosis through histological analysis. Ang II infusion resulted in an increased size and disorganised myofibers in heart tissues. Wheat germ agglutinin (WGA) staining of the sections showed significant cardiomyocyte hypertrophy in Ang II‐treated mice (Figure 2C). Staining of heart tissues with Masson's and Sirius red also showed that Ang II caused significant collagen deposition in heart tissues (Figure 2D,E). All these changes were not altered in the MyD88^f/f^ Myh6‐Cre + Ang II group (Figure 2A–E, Figure S2D). This was further confirmed by RT‐qPCR analysis of key mediators of fibrogenic genes (*Col1a1* and *Tgfb*) and hypertrophy (*Myh7*) (Figure S2F). Since chronic inflammation is extremely essential in cardiac remodelling and dysfunction, we also detected cardiac inflammation by determining macrophage infiltration and inflammatory cytokines expression. As shown in Figure 2F, Ang II infusion caused a significant increase in macrophage infiltration, whereas cardiac deletion of MyD88 did not attenuate the situation. RT‐qPCR analysis of canonical inflammatory cytokines (*Il1b*, *Il6* and *Tnfa*) also confirmed the point. Taken together, these results indicated that cardiomyocyte‐specific MyD88 knockout shows no significant protection in Ang II‐induced cardiac hypertrophy.

### 3.3 Macrophage‐Specific Knockout of MyD88 Attenuates Ang II‐Induced Cardiac Hypertrophy

**FIGURE 3:**
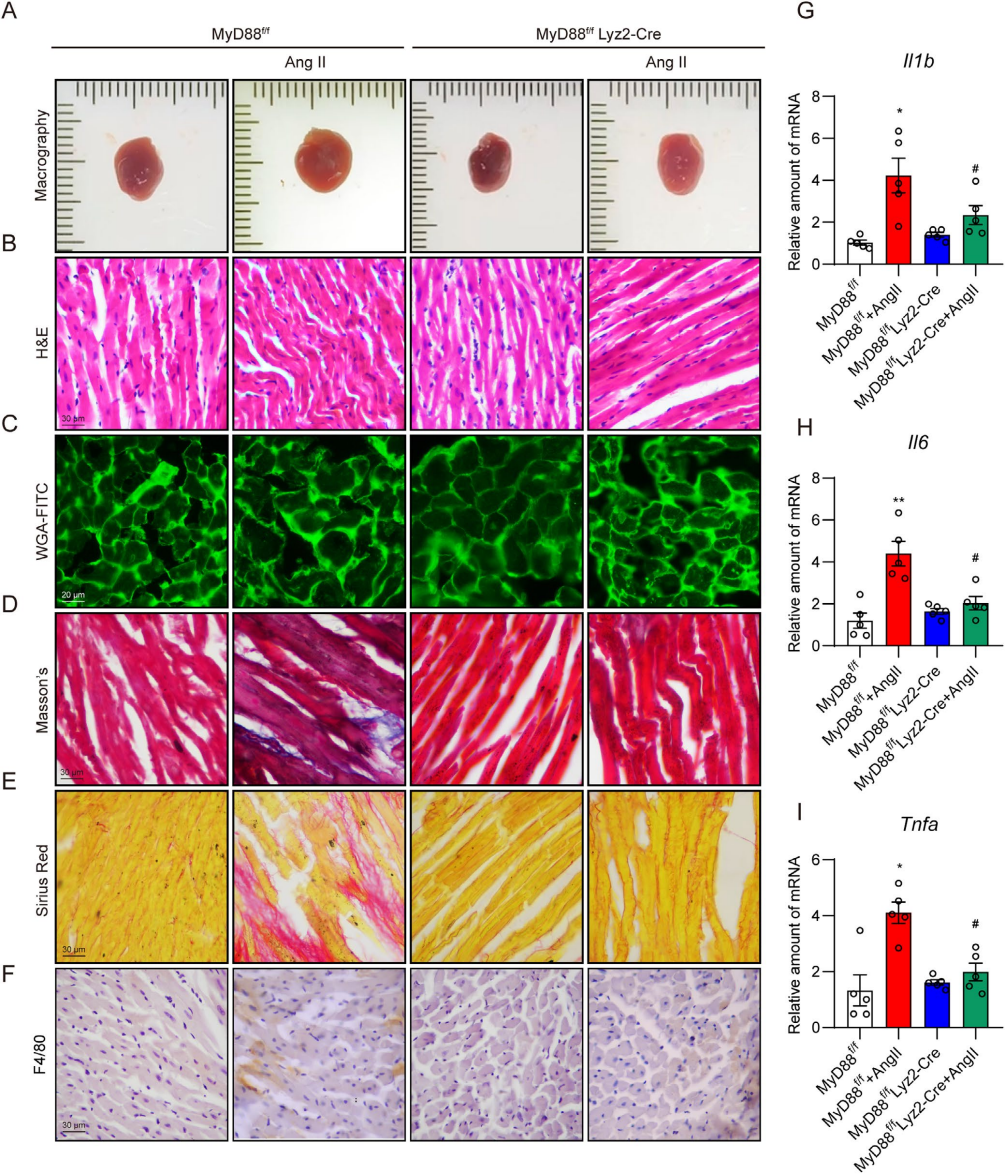
Macrophage‐specific knockout of MyD88 attenuates Ang II‐induced cardiac hypertrophy. MyD88^f/f^ and MyD88^f/f^ Lyz2‐Cre mice were continuously challenged with Ang II (1 μg/kg/min) or normal saline using micro‐osmotic pumps for 4 weeks. (A) Representative images of heart tissues. (B) Haematoxylin & Eosin staining showing myocardial fibre arrangements in heart tissues [scale bar = 30 μm]. (C) WGA‐FITC staining showing the cross‐sectional area of cardiomyocytes in heart tissues [scale bar = 20 μm]. (D) Masson's staining showing the interstitial fibrotic area in heart tissues [scale bar = 30 μm]. (E) Sirius Red staining showing the heart tissue fibres [scale bar = 30 μm]. (F) Anti‐F4/80 staining showing macrophage infiltration in heart tissues [scale bar = 30 μm]. (G–I) RT‐qPCR analysis of *Il1b* (G), *Il6* (H), and *Tnfa* (I) in heart tissues. Data were presented as mean ± SEM, *n* = 5; *p < 0.05; **p < 0.01, compared to the MyD88^f/f^ group; ^#^ *p* < 0.05 compared to the MyD88^f/f^ + Ang II group.

Then, we further explored the role of MyD88 in macrophages in the development of Ang II‐induced cardiac remodelling and dysfunction. We crossed MyD88^f/f^ mice with the macrophage‐specific Cre mouse (Lyz2‐Cre) to obtain MyD88^f/f^ Lyz2‐Cre mice, selectively eliminating MyD88 expression in macrophages. After the Ang II infusion, we found that MyD88 macrophage‐specific knockout did not affect body weight, systolic blood pressure, and serum Ang II levels (Figure S3A–C). Non‐invasive echocardiography analysis revealed that compared to the MyD88^f/f^ + Ang II group, mice in the MyD88^f/f^ Lyz2‐Cre + Ang II group showed significant improvements in cardiac systolic function, evidenced by increased EF and FS (Table 2). Parameters of the cardiac structure also indicated that MyD88 macrophage‐specific knockout reduced Ang II‐induced cardiac remodelling, which was confirmed by decreased HW/BW and HW/TL (Table 2). We also observed that cardiac injury was improved in the MyD88^f/f^ Lyz2‐Cre + Ang II group, as serum markers such as CK‐MB and BNP were revealed (Table 2, Figure S3E). Besides, histological analysis showed that both cardiac fibrosis and cardiomyocyte hypertrophy were significantly improved in MyD88^f/f^ Lyz2‐Cre mice treated with Ang II (Figure 3A–E, Figure S3D–F). Moreover, cardiac inflammation was also attenuated in MyD88^f/f^ Lyz2‐Cre mice treated with Ang II (Figure 3F–I). Collectively, these data demonstrated that MyD88 macrophage‐specific knockout attenuates Ang II‐induced cardiac remodelling and dysfunction.

**TABLE 2:** Biometric and echocardiographic parameters of the Model 2 experimental mice.

Parameters	MyD88^f/f^ (*n* = 5)	MyD88^f/f^ (*n* = 5)	MyD88^f/f^ Lyz2‐Cre (*n* = 5)	MyD88^f/f^ Lyz2‐Cre (*n* = 5)
		Ang II (pump)		Ang II (pump)
Heart rate, bpm	460.47 ± 11.38	465.59 ± 3.26	458.15 ± 3.48	461.04 ± 2.51
EF, %	66.73 ± 1.38	43.68 ± 2.58*	64.7 ± 2.28	61.9 ± 3.63^#^
FS, %	36.54 ± 1.02	21.32 ± 1.44*	34.99 ± 1.59	33.03 ± 2.57^#^
LVAWs, mm	1.34 ± 0.04	1.46 ± 0.08*	1.25 ± 0.05	1.38 ± 0.05^#^
LVAWd, mm	0.79 ± 0.05	1.05 ± 0.04*	0.88 ± 0.05	0.95 ± 0.05^#^
LVPWs, mm	1.16 ± 0.04	1.14 ± 0.03	1.22 ± 0.04	1.17 ± 0.06
LVPWd, mm	0.66 ± 0.04	0.96 ± 0.04*	0.68 ± 0.02	0.74 ± 0.03^#^
Weight, g	25.11 ± 0.32	23.75 ± 0.36	23.42 ± 0.55	23.66 ± 0.92
HW/BW, mg/g	5.40 ± 0.15	7.42 ± 0.23*	5.76 ± 0.07	5.87 ± 0.19^#^
HW/TL, mg/cm	7.40 ± 0.17	9.65 ± 0.08*	7.68 ± 0.04	7.75 ± 0.18^#^
CK‐MB, U/L	163.13 ± 9.22	305.74 ± 15.13*	175.73 ± 14.38	189.89 ± 17.23^#^
BNP, μg/L	0.11 ± 0.02	0.29 ± 0.02*	0.11 ± 0.02	0.13 ± 0.02^#^

*Note:* *p < 0.05, compared to MyD88^f/f^ group; ^#^ *p* < 0.05, compared to MyD88^f/f^ + Ang II group.

### 3.4 Pharmacological Inhibition of MyD88 Ameliorates Ang II‐Induced Cardiac Hypertrophy

**FIGURE 4:**
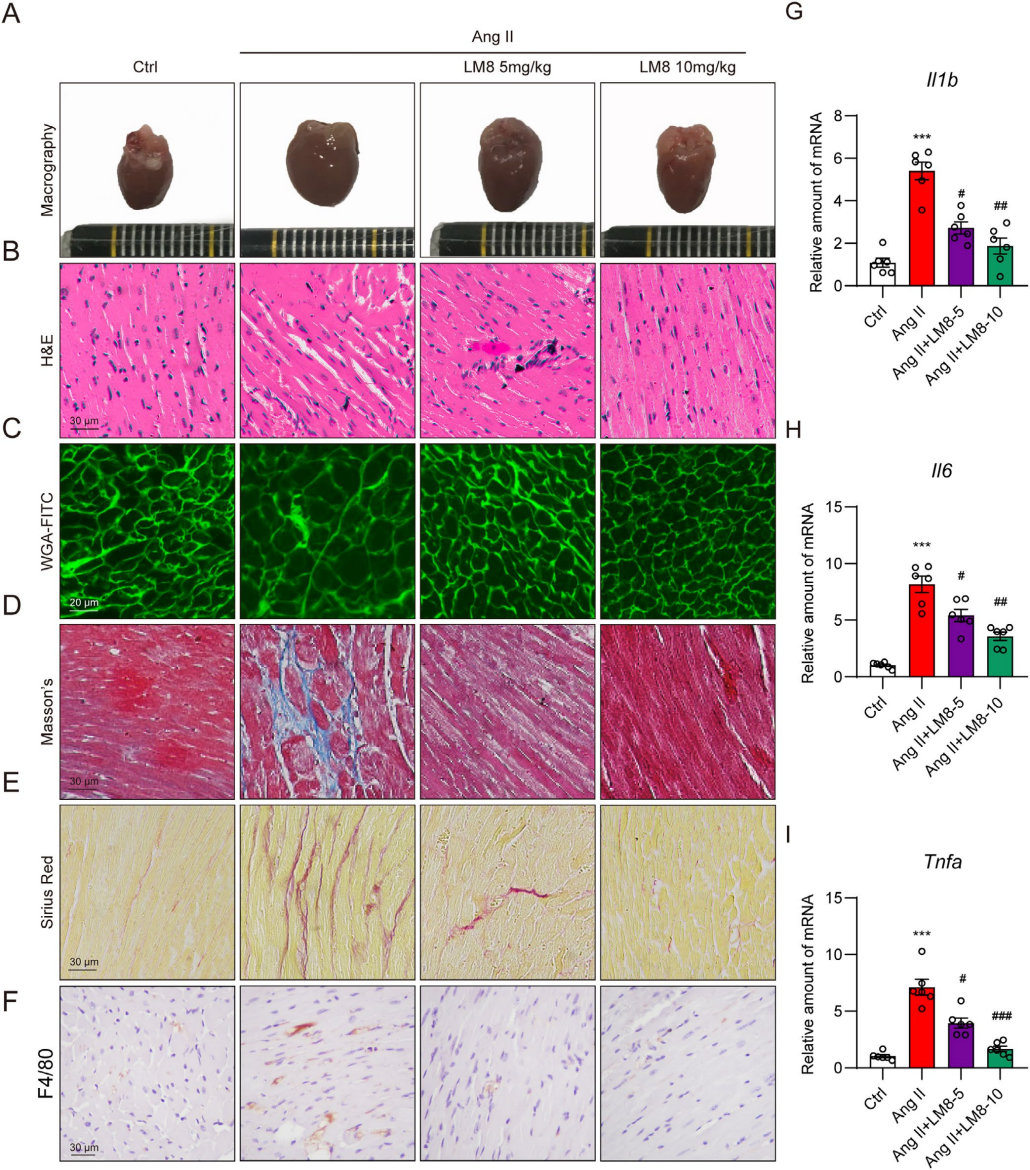
MyD88 inhibitor ameliorates Ang II‐induced cardiac hypertrophy in vivo. C57BL/6 mice were continuously challenged with Ang II (1 μg/kg/min) using micro‐osmotic pumps for 4 weeks. Three weeks after Ang II infusion, mice were administered with MyD88 inhibitor, LM8 (5 or 10 mg/kg), or vehicle control (CMC‐Na) via oral gavage daily for 2 weeks. (A) Representative images of heart tissues. (B) Haematoxylin & Eosin staining showing myocardial fibre arrangements in heart tissues [scale bar = 30 μm]. (C) WGA‐FITC staining showing the cross‐sectional area of cardiomyocytes in heart tissues [scale bar = 20 μm]. (D) Masson's staining showing the interstitial fibrotic area in heart tissues [scale bar = 30 μm]. (E) Sirius Red staining showing the heart tissue fibres [scale bar = 30 μm]. (F) Anti‐F4/80 staining showing macrophage infiltration in heart tissues [scale bar = 30 μm]. (G–I) RT‐qPCR analysis of *Il1b* (G), *Il6* (H) and *Tnfa* (I) in heart tissues. Data were presented as mean ± SEM, *n* = 6; ***p < 0.001, compared to the Ctrl group; ^#^ *p* < 0.05; ^##^ *p* < 0.01; ^###^ *p* < 0.001, compared to the Ang II group.

We then determined whether MyD88 inhibition offers similar protection in the Ang II‐induced hypertrophy model. LM8, synthesised previously in our lab [20], was selected as the pharmacological inhibitor of MyD88. LM8 dissolved in 0.5% CMC‐Na was administered by gavage 2 weeks after Ang II infusion and maintained for 2 weeks. As expected, although the administration of LM8 did not influence body weight, systolic blood pressure, and serum Ang II levels (Figure S4A–C), it significantly improved cardiac function as demonstrated by non‐invasive echocardiography (Table 3, Figure S4E). Other parameters, such as HW/BW, HW/TL and serum markers of cardiac injury, further confirmed this (Table 3). Histological analysis showed that LM8 exhibited dose‐dependent protective effects against Ang II‐induced cardiac fibrosis, cardiomyocyte hypertrophy, as well as cardiac inflammation (Figure 4, Figure S4).

**TABLE 3:** Biometric and echocardiographic parameters of the Model 3 experimental mice.

Parameters	WT (*n* = 6)	Ang II (pump)		
		WT (*n* = 7)	LM8 (5 mg/kg) (*n* = 6)	LM8 (10 mg/kg) (*n* = 6)
Heart rate, bpm	438.1 ± 16.46	427.46 ± 11.85	419.69 ± 11.81	427.41 ± 11.62
EF, %	73.34 ± 3.02	54.31 ± 2.77***	68.52 ± 2.36^##^	72.04 ± 2.53^###^
FS, %	42.11 ± 2.66	27.78 ± 1.79**	37.85 ± 1.91^#^	40.54 ± 2.12^##^
LVAWs, mm	1.45 ± 0.11	1.59 ± 0.12	1.58 ± 0.14	1.66 ± 0.15
LVAWd, mm	0.78 ± 0.12	1.15 ± 0.15	0.99 ± 0.13	1.03 ± 0.13
LVPWs, mm	1.32 ± 0.09	1.1 ± 0.09	1.43 ± 0.09	1.47 ± 0.09
LVPWd, mm	0.76 ± 0.07	0.82 ± 0.06	1.04 ± 0.1	1.03 ± 0.11
Weight, g	23.72 ± 0.5	23.68 ± 0.98	23.83 ± 0.97	23.23 ± 0.74
HW/BW, mg/g	5.42 ± 0.19	6.55 ± 0.14*	5.98 ± 0.33^#^	5.87 ± 0.24^#^
HW/TL, mg/cm	6.98 ± 0.27	8.51 ± 0.33*	7.77 ± 0.43^#^	7.22 ± 0.18^#^
CK‐MB, U/L	136.73 ± 15.97	361.56 ± 8.94*	185.26 ± 9.081^#^	135.07 ± 10.708^#^
BNP. μg/L	0.12 ± 0.004	0.22 ± 0.012*	0.16 ± 0.007	0.15 ± 0.008^#^

*Note:* *p < 0.05; **p < 0.01; ***p < 0.001 compared to WT group; ^#^ *p* < 0.05; ^##^ *p* < 0.01; ^###^ *p* < 0.001 compared to WT + Ang II group.

### 3.5 MyD88 Mediates Ang II‐Induced Macrophage Infiltration by Regulating Chemokine Secretion

**FIGURE 5:**
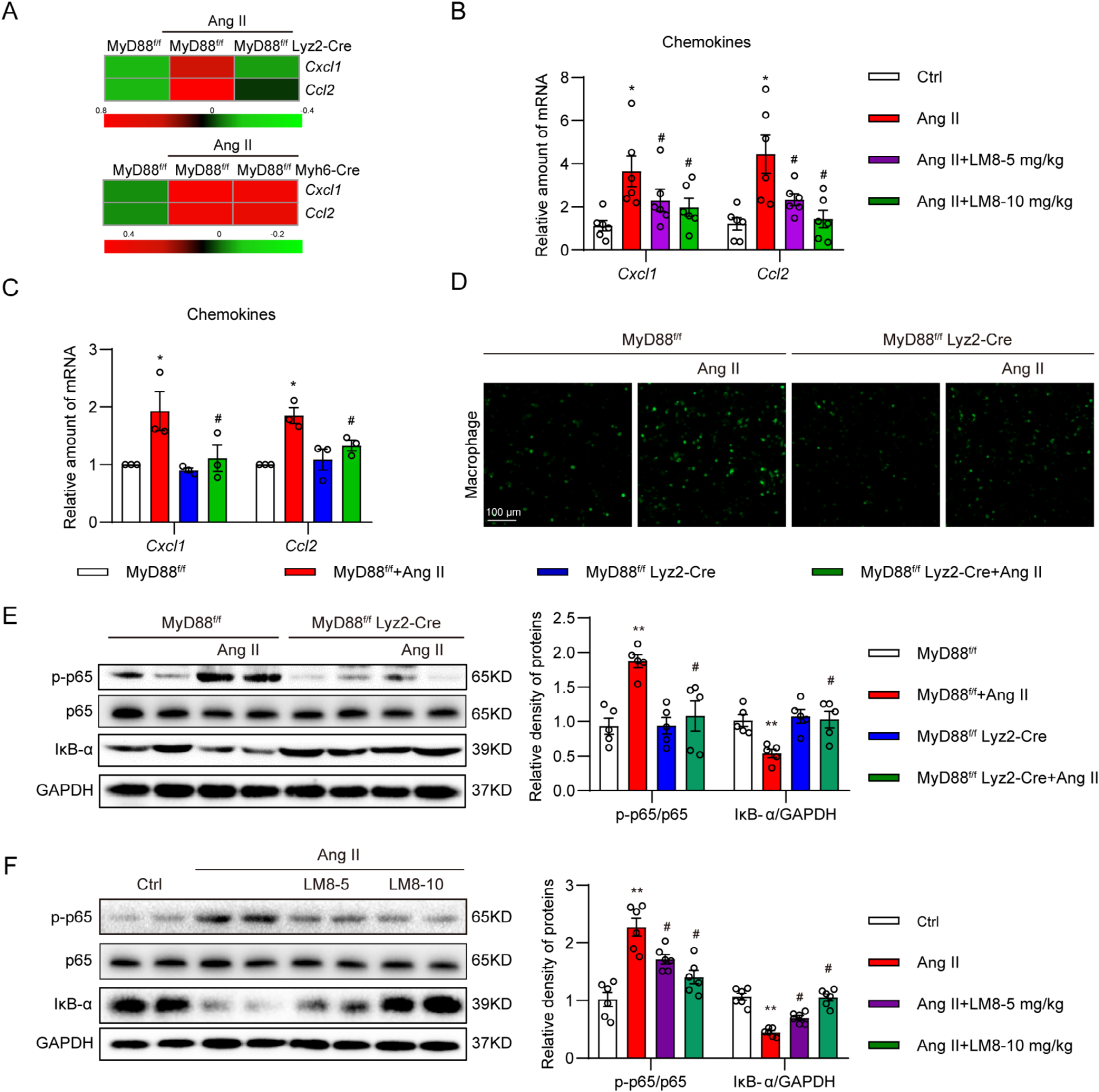
MyD88 mediates Ang II‐induced macrophage infiltration via regulating chemokine secretion. (A) RT‐qPCR analysis of inflammatory chemokines and cytokines in heart tissues from MyD88 cardiac‐specific knockout (upper panel) and MyD88 macrophage‐specific knockout mice (lower panel) with or without Ang II treatment. (B) RT‐qPCR validation of *Cxcl1* and *Ccl2* mRNA levels in heart tissues of Ang II‐challenged mice with or without LM8 treatment. (C) RT‐qPCR analysis of *Cxcl1* and *Ccl2* mRNA levels in MyD88‐deficient macrophages challenged with 1 μM Ang II for 6 h. (D) Macrophage adhesion experiments. Macrophages derived from MyD88^f/f^ Lyz2‐Cre mice were incubated with CFSE for 30 min, followed by stimulation with 1 μM Ang II for 6 h. The stimulated MyD88‐deficient macrophages were added to the cultured H9c2 cells for 4 h. The non‐attached macrophages were washed three times with PBS. The attached macrophages were measured by detecting CFSE immunofluorescence [scale bar = 100 μm]. (E) Representative Western blot images of the NF‐κB pathway in cardiac tissues from MyD88^f/f^ Lyz2‐Cre mice infused with Ang II. The right panel shows the densitometric quantifications. (F) Representative Western blot images of the NF‐κB pathway in cardiac tissues from LM8‐treated C57BL/6 mice infused with Ang II. The right panel shows the densitometric quantifications. Statistical data were presented as mean ± SEM, *n* = 5–6 for in vivo data, *n* = 3–5 for in vitro data; *p < 0.05; **p < 0.01, compared to the Ctrl group; ^#^ *p* < 0.05 compared to the Ang II‐treated group.

Mechanically, we evaluated how MyD88 affects macrophage infiltration and cardiac function readouts. Firstly, we measured the transcription of inflammatory cytokines and chemokines using qPCR analysis. Similar to the results in Figures 2 and 3, cardiomyocyte‐specific MyD88 knockout mice and macrophage‐specific MyD88 knockout mice showed totally different cardiac transcription changes upon Ang II infusion in *Myh7*, *Il6*, *Il1b*, *Tnfa* and *Cd68* (Figure S5A). As shown in Figure S5A, mRNA levels of *Ccl3, Ccl4*, *Ccl5*, *Cxcl24*, *Cxcl9*, *Cxcl12*, *Il33* and *Socs3* remained unchanged between Ang II‐treated MyD88^f/f^ mice and Ang II‐treated MyD88^f/f^ Lyz2‐Cre mice. Similar results were found in Ang II‐treated MyD88^f/f^ mice and Ang II‐treated MyD88^f/f^ Myh6‐Cre mice (Figure S5B). However, transcription of *Cxcl1* and *Ccl2* was upregulated in Ang II‐treated MyD88^f/f^ mice, which was markedly downregulated in Ang II‐treated MyD88^f/f^ Lyz2‐Cre mice, whereas they remained high in MyD88^f/f^ Myh6‐Cre mice (Figure 5A). RT‐qPCR analysis of these two genes in LM8‐treated mice showed similar results (Figure 5B). Besides, primary macrophages derived from MyD88^f/f^ and MyD88^f/f^ Lyz2‐Cre mice treated with 1 μM Ang II confirmed that MyD88 deletion in macrophages was associated with the reduction of chemokines *Cxcl1* and *Ccl2* (Figure 5C). In vitro adhesion experiment also demonstrated that Ang II could significantly increase the infiltration of MyD88^f/f^ mice‐derived macrophages, but failed in macrophages from MyD88^f/f^ Lyz2‐Cre mice (Figure 5D, Figure S6). Since these chemokines were mainly regulated by NF‐κB signalling, we then measured the NF‐κB activity in MyD88^f/f^ Lyz2‐Cre hypertensive mice and LM8‐treated hypertensive mice. As expected, macrophage‐specific MyD88 knockout and MyD88 inhibitor significantly suppressed the activation of NF‐κB signalling, evidenced by reduced p‐p65 and increased IκB‐α levels (Figure 5E,F). Together, these data demonstrated that MyD88 mediates Ang II‐induced macrophage infiltration via regulating chemokine secretion.

### 3.6 Conditioned Medium From MyD88‐Deficient Macrophages Failed to Induce Cardiomyocyte Hypertrophy

**FIGURE 6:**
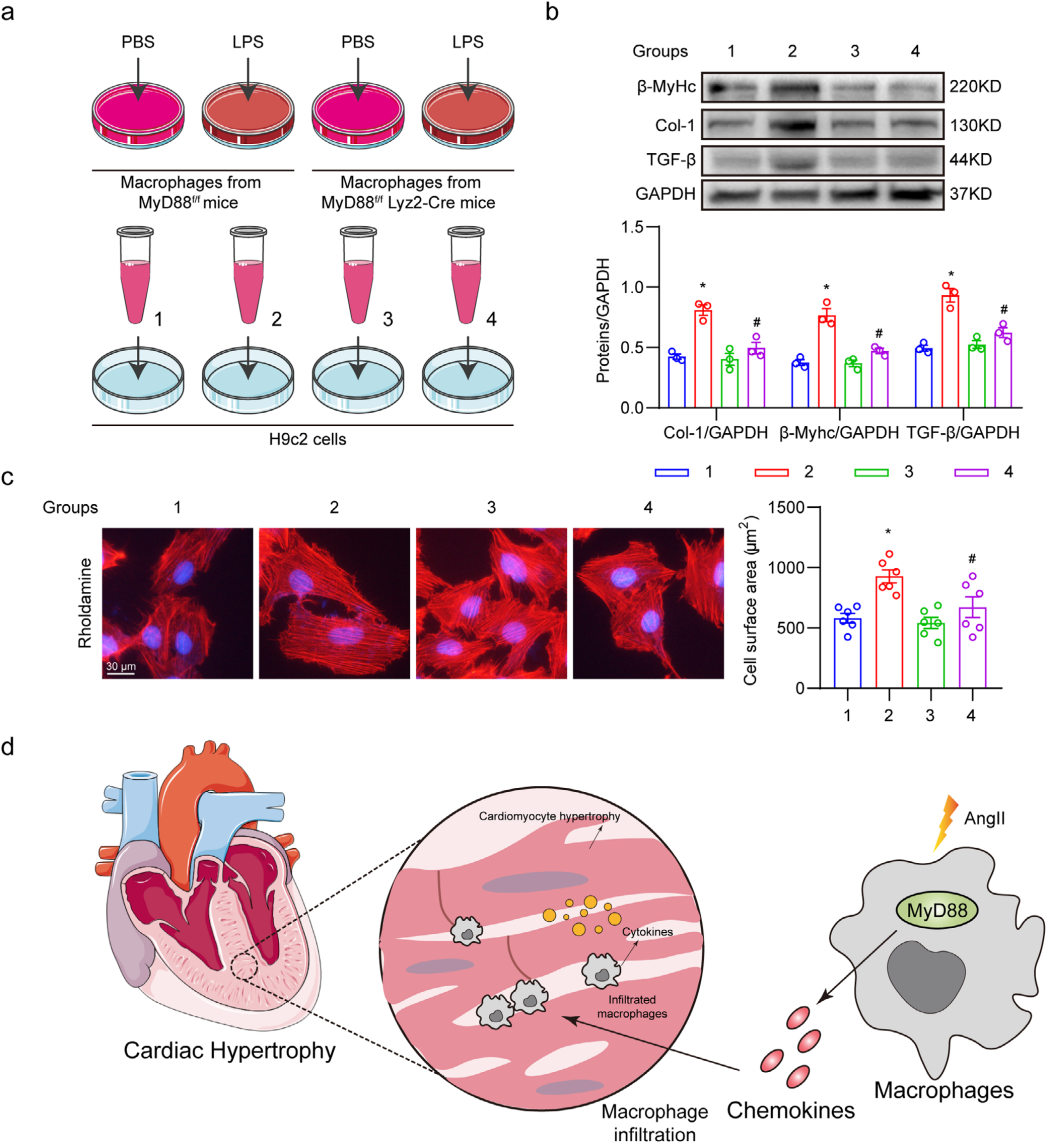
Conditioned medium from MyD88‐deficient macrophages fails to induce cardiomyocyte hypertrophy. (a) Schematic illustration of the experimental design of the conditioned medium study. Primary macrophages derived from MyD88^f/f^ and MyD88^f/f^ Lyz2‐Cre mice were treated with LPS (50 μg/mL) or PBS for 24 h. The cell culture medium was collected as the conditioned medium. H9c2 cells were treated with different conditioned medium for 24 h. (b) The protein levels of β‐Myhc, Col‐1, and TGF‐β in primary cardiomyocytes were evaluated using Western blot analysis. Representative images (upper panel) and the densitometric quantifications (lower panel) are shown. (c) Representative images of Rhodamine phalloidin staining in H9c2 cells [scale bar = 30 μm] (left panel). The statistical quantification of cellular hypertrophy was shown (right panel). (d) Schematic demonstrating mechanisms involved in Myeloid MyD88 mediates macrophage infiltration and activation in Ang II‐induced cardiac hypertrophy. Statistical data were presented as mean ± SEM, *n* = 3 for in vitro data; *p < 0.05 compared to group 1; ^#^ *p* < 0.05 compared to group 2.

Inflammatory cytokines bridge the infiltrated macrophages and cardiac injury, contributing to cardiac remodelling and negatively affecting cardiomyocytes. Also, we found that canonical inflammatory cytokines were significantly reduced in MyD88^f/f^ Lyz2‐Cre mice and LM8‐treated mice (Figures 3G–I and 4G–I). To mimic the situation in vitro, we performed the conditioned medium experiments (Figure 6a). LPS‐treated macrophages were utilised as activated macrophages. Conditioned medium from 4 groups was added to the cultured medium of H9c2 cells to stimulate cellular hypertrophy. Western blot analysis showed that conditioned medium from MyD88‐deficient macrophages (group 4) failed to increase the expression of cellular hypertrophy and fibrosis markers, whereas the conditioned medium from MyD88^f/f^ mice (group 2) significantly upregulated the profiles (Figure 6b). This was further confirmed by Rhodamine‐phalloidin staining (Figure 6c). Taken together, these results indicate that MyD88 blockade in macrophages reduces the inflammatory cytokine secretion and protects cardiomyocytes.

## 4 Discussion

In this study, we demonstrated that MyD88 in myeloid cells, rather than cardiomyocytes, contributed to Ang II‐induced cardiac inflammation and fibrosis. MyD88‐specific inhibitor LM8 offered similar protection. Macrophage deletion of MyD88 reduced secretion of CXCL1 and CCL2, which might be the mechanism underlying decreased macrophage infiltration in vivo. Moreover, we found that these activated macrophages could further induce cardiomyocyte hypertrophy in vitro, whereas the deletion of MyD88 in macrophages suppressed this process. Therefore, myeloid MyD88 could be a potential target for treating hypertensive cardiac injury. A summary of our main findings is presented in Figure 6d.

At least three groups have reported the effects of MyD88 knockout in the Ang II‐induced hypertensive mouse model: Singh et al. [26], Wang et al. [27], and Owens et al. [28]. Controversially, Singh et al. [26] find that MyD88 deficiency further increases blood pressure and aggravates Ang II‐induced cardiac hypertrophy. In contrast, both Wang et al. [27] and Owens et al. [28] show that MyD88 knockout significantly alleviates Ang II‐induced cardiac function, hypertrophy, and atherosclerosis. In addition, Ha et al. [29] report that MyD88 knockdown attenuates pressure overload‐induced cardiac hypertrophy and decreases cardiomyocyte apoptosis in mice. Therefore, MyD88 may play a dual role in Ang II‐induced cardiovascular pathologies, where it can be either beneficial or detrimental. Thus, it is very necessary and significant to examine the effects of MyD88 in Ang II‐induced models. In our study, we found that although MyD88 deficiency in cardiomyocytes showed no significant protective effects, MyD88 deficiency in macrophages and systemic administration of the MyD88‐specific inhibitor, LM8, could ameliorate cardiac inflammation and fibrosis without affecting blood pressure. In addition, we noted that in the study of Singh et al. [26], Ang II infusion concentration is 3000 ng/kg per minute, much higher than 1000 ng/kg per minute used in the studies of Wang et al. [27] and our group. It needs to be further investigated if MyD88 plays a beneficial role in an extremely high‐concentration Ang II condition.

Furthermore, recent studies elucidated the role of MyD88 in specific cell types during cardiac remodelling. Abraham et al. [30] demonstrated that T cell‐specific MyD88 regulates T cell activation and survival, thereby promoting inflammation and cardiac fibrosis following transverse aortic constriction. Similarly, Umbarkar et al. [31] reported that fibroblast‐specific knockout of MyD88 alleviates myocardial infarction‐induced cardiac dysfunction and remodelling. In our study, we demonstrated that MyD88 in macrophages facilitates their infiltration and contributes to cardiac inflammation and remodelling. Emerging evidence has emphasised the role of immune cells in developing cardiac hypertrophy [32]. Among the immune cells, macrophages are extensively explored in hypertension‐associated cardiac injury. On the one hand, inflammatory cytokines secreted by macrophages elevate blood pressure by triggering vascular endothelial dysfunction [33]. Wenzel et al. [34] demonstrate that depletion of lysozyme M^+^ (LysM^+^) macrophages limits blood pressure elevation. On the other hand, activated macrophages exert a strong release of prototypical M1 macrophage cytokines, including TNF‐α and IL‐1β [35]. Subsequently, these pro‐inflammatory cytokines further induce tissue damage via multiple signalling pathways, including MAPK, TGF‐β and NF‐κB [36]. Besides, macrophage infiltration also plays an important role in hypertensive injury. In CCR2‐deficient mice, Ang II‐induced vascular inflammation and remodelling are significantly blunted compared to control mice [37]. Antagonising CX3CR1 markedly reduces the development of cardiac hypertrophy [38]. Wang et al. [12] also demonstrate that the CXCL1/CXCR2 axis mediates Ang II‐induced monocyte infiltration and contributes to cardiac hypertrophy. In our study, although deletion of MyD88 in macrophages did not affect blood pressure, it decreased prototypical M1 macrophage‐derived cytokines in vivo and reduced chemokines of CXCL1 and CCL2. Macrophage‐specific knockout of MyD88 also suppressed their activation and inhibited conditioned medium‐induced cardiomyocyte hypertrophy.

It should be noted that there is sufficient evidence supporting that MyD88 mediates the secretion of multiple chemokines. Deficiency of MyD88 shows lower levels of CCL5, CXCL10, CCL3, and CXCL13 in the mouse serum [39]. Mühlbauer et al. [40] also report that epithelial cell‐specific MyD88 mediates expression of CCL2 and CXCL1 in ischaemia/reperfusion‐induced intestinal injury in mice. Besides, MyD88 is also reported to regulate the expression of other chemokines, such as CCL2, CCL7, CXCL2, CXCL3, CCL8 and CXCL9 in LPS‐treated macrophages [41]. In our study, we utilised a screening for several chemokines in MyD88^f/f^ Myh6‐Cre mice and MyD88^f/f^ Lyz2‐Cre mice. We found that CXCL1 and CCL2 were differentially expressed and might be associated with different macrophage infiltration in heart tissue. We further confirmed that NF‐κB signalling could be the potential pathway linking MyD88 and CXCL1/CCL2 expression, which was consistent with the previous findings [42, 43, 44].

There are also some limitations in our study, including the lack of flow cytometry analysis for infiltrated immune cells and the absence of a category for different macrophages in hypertensive hearts. Besides, high‐throughput sequencing is further needed to fully reveal the difference between Ang II‐treated macrophages derived from MyD88‐deficient and cardiomyocyte‐deficient mice.

## 5 Conclusions

In summary, our study first shows that myeloid MyD88 rather than cardiomyocyte MyD88 contributes to Ang II‐induced cardiac remodelling. We demonstrate that macrophage MyD88 mediates Ang II‐induced chemokine secretion and macrophage infiltration in heart tissues, further activating macrophages and promoting the hypertrophy phenotype of cardiomyocytes. Targeting MyD88 therapy might be a potential strategy for hypertensive cardiac remodelling.

## Author Contributions

**Ke Lin:** data curation (equal), investigation (equal), methodology (equal), writing – original draft (equal). **Na Yang:** data curation (equal), investigation (equal), methodology (equal). **Chenghong Hu:** data curation (equal), methodology (equal). **Wu Luo:** methodology (equal), supervision (equal). **Ping Huang:** investigation (equal), methodology (equal). **Guang Liang:** data curation (equal), methodology (equal), supervision (equal). **Yi Wang:** investigation (equal), methodology (equal), resources (equal), validation (equal), writing – review and editing (equal).

## Conflicts of Interest

The authors declare no conflicts of interest.

## Supporting information


Data S1.

## Data Availability

The data that support the findings of this study are available from the corresponding author upon reasonable request.

## References

[1] S. Oparil , M. C. Acelajado , G. L. Bakris , et al., “Hypertension,” Nature Reviews Disease Primers 4 (2018): 18014, 10.1038/nrdp.2018.14. PMC6477925 29565029

[2] T. A. McDonagh , M. Metra , M. Adamo , et al., “ESC Guidelines for the Diagnosis and Treatment of Acute and Chronic Heart Failure,” European Heart Journal 42, no. 36 (2021): 3599–3726, 10.1093/eurheartj/ehab368. 34447992

[3] S. J. Forrester , G. W. Booz , C. D. Sigmund , et al., “Angiotensin II Signal Transduction: An Update on Mechanisms of Physiology and Pathophysiology,” Physiological Reviews 98, no. 3 (2018): 1627–1738, 10.1152/physrev.00038.2017. 29873596 PMC6335102

[4] L. B. Arendse , A. H. J. Danser , M. Poglitsch , et al., “Novel Therapeutic Approaches Targeting the Renin‐Angiotensin System and Associated Peptides in Hypertension and Heart Failure,” Pharmacological Reviews 71, no. 4 (2019): 539–570, 10.1124/pr.118.017129. 31537750 PMC6782023

[5] A. C. Montezano , A. Nguyen Dinh Cat , F. J. Rios , and R. M. Touyz , “Angiotensin II and Vascular Injury,” Current Hypertension Reports 16, no. 6 (2014): 431, 10.1007/s11906-014-0431-2. 24760441

[6] G. R. Drummond , A. Vinh , T. J. Guzik , and C. G. Sobey , “Immune Mechanisms of Hypertension,” Nature Reviews Immunology 19, no. 8 (2019): 517–532, 10.1038/s41577-019-0160-5. 30992524

[7] J. Wu , E. Dong , Y. Zhang , and H. Xiao , “The Role of the Inflammasome in Heart Failure,” Frontiers in Physiology 12 (2021): 709703, 10.3389/fphys.2021.709703. 34776995 PMC8581560

[8] J. Ye , L. Liu , Q. Ji , et al., “Anti‐Interleukin‐22‐Neutralizing Antibody Attenuates Angiotensin II‐Induced Cardiac Hypertrophy in Mice,” Mediators of Inflammation 2017 (2017): 5635929, 10.1155/2017/5635929. 29358851 PMC5735629

[9] J. Han , S. Ye , C. Zou , et al., “Angiotensin II Causes Biphasic STAT3 Activation Through TLR4 to Initiate Cardiac Remodeling,” Hypertension 72, no. 6 (2018): 1301–1311, 10.1161/hypertensionaha.118.11860. 30571233

[10] T. J. Guzik , N. E. Hoch , K. A. Brown , et al., “Role of the T Cell in the Genesis of Angiotensin II Induced Hypertension and Vascular Dysfunction,” Journal of Experimental Medicine 204, no. 10 (2007): 2449–2460, 10.1084/jem.20070657. 17875676 PMC2118469

[11] Y. N. Nabah , T. Mateo , R. Estellés , et al., “Angiotensin II Induces Neutrophil Accumulation In Vivo Through Generation and Release of CXC Chemokines,” Circulation 110, no. 23 (2004): 3581–3586, 10.1161/01.Cir.0000148824.93600.F3. 15569833

[12] L. Wang , Y. L. Zhang , Q. Y. Lin , et al., “CXCL1‐CXCR2 Axis Mediates Angiotensin II‐Induced Cardiac Hypertrophy and Remodelling Through Regulation of Monocyte Infiltration,” European Heart Journal 39, no. 20 (2018): 1818–1831, 10.1093/eurheartj/ehy085. 29514257

[13] Y. N. Abu Nabah , M. Losada , R. Estellés , et al., “CXCR2 Blockade Impairs Angiotensin II‐Induced CC Chemokine Synthesis and Mononuclear Leukocyte Infiltration,” Arteriosclerosis, Thrombosis, and Vascular Biology 27, no. 11 (2007): 2370–2376, 10.1161/atvbaha.107.147009. 17717298

[14] L. Chen , L. Zheng , P. Chen , and G. Liang , “Myeloid Differentiation Primary Response Protein 88 (MyD88): The Central Hub of TLR/IL‐1R Signaling,” Journal of Medicinal Chemistry 63, no. 22 (2020): 13316–13329, 10.1021/acs.jmedchem.0c00884. 32931267

[15] J. Deguine and G. M. Barton , “MyD88: A Central Player in Innate Immune Signaling,” F1000prime Reports 6 (2014): 97, 10.12703/p6-97. 25580251 PMC4229726

[16] W. Gao , H. Wang , L. Zhang , et al., “Retinol‐Binding Protein 4 Induces Cardiomyocyte Hypertrophy by Activating TLR4/MyD88 Pathway,” Endocrinology 157, no. 6 (2016): 2282–2293, 10.1210/en.2015-2022. 27100622 PMC4891784

[17] J. Han , C. Zou , L. Mei , et al., “MD2 Mediates Angiotensin II‐Induced Cardiac Inflammation and Remodeling via Directly Binding to Ang II and Activating TLR4/NF‐κB Signaling Pathway,” Basic Research in Cardiology 112, no. 1 (2017): 9, 10.1007/s00395-016-0599-5. 28013347

[18] M. V. Singh , M. Z. Cicha , S. Nunez , D. K. Meyerholz , M. W. Chapleau , and F. M. Abboud , “Angiotensin II‐Induced Hypertension and Cardiac Hypertrophy Are Differentially Mediated by TLR3‐ and TLR4‐Dependent Pathways,” American Journal of Physiology Heart and Circulatory Physiology 316, no. 5 (2019): H1027–H1038, 10.1152/ajpheart.00697.2018. 30793936 PMC6580398

[19] S. Ye , K. Lin , G. Wu , et al., “Toll‐Like Receptor 2 Signaling Deficiency in Cardiac Cells Ameliorates Ang II‐Induced Cardiac Inflammation and Remodeling,” Translational Research 233 (2021): 62–76, 10.1016/j.trsl.2021.02.011. 33652137

[20] L. Chen , H. Chen , P. Chen , et al., “Development of 2‐Amino‐4‐Phenylthiazole Analogues to Disrupt Myeloid Differentiation Factor 88 and Prevent Inflammatory Responses in Acute Lung Injury,” European Journal of Medicinal Chemistry 161 (2019): 22–38, 10.1016/j.ejmech.2018.09.068. 30342423

[21] T. Jin , J. Lin , Y. Gong , et al., “iPLA(2)β Contributes to ER Stress‐Induced Apoptosis During Myocardial Ischemia/Reperfusion Injury,” Cells 10, no. 6 (2021): 1446, 10.3390/cells10061446. 34207793 PMC8227999

[22] S. Ye , W. Luo , Z. A. Khan , et al., “Celastrol Attenuates Angiotensin II‐Induced Cardiac Remodeling by Targeting STAT3,” Circulation Research 126, no. 8 (2020): 1007–1023, 10.1161/circresaha.119.315861. 32098592

[23] Y. Zhang , T. Xu , Z. Pan , et al., “Shikonin Inhibits Myeloid Differentiation Protein 2 to Prevent LPS‐Induced Acute Lung Injury,” British Journal of Pharmacology 175, no. 5 (2018): 840–854, 10.1111/bph.14129. 29243243 PMC5811619

[24] V. Ravi , A. Jain , A. Taneja , K. Chatterjee , and N. R. Sundaresan , “Isolation and Culture of Neonatal Murine Primary Cardiomyocytes,” Current Protocols 1, no. 7 (2021): e196, 10.1002/cpz1.196. 34289259

[25] P. G. Hitscherich , L. H. Xie , D. Del Re , and E. J. Lee , “The Effects of Macrophages on Cardiomyocyte Calcium‐Handling Function Using In Vitro Culture Models,” Physiological Reports 7, no. 13 (2019): e14137, 10.14814/phy2.14137. 31301118 PMC6640591

[26] M. V. Singh , M. Z. Cicha , D. K. Meyerholz , M. W. Chapleau , and F. M. Abboud , “Dual Activation of TRIF and MyD88 Adaptor Proteins by Angiotensin II Evokes Opposing Effects on Pressure, Cardiac Hypertrophy, and Inflammatory Gene Expression,” Hypertension 66, no. 3 (2015): 647–656, 10.1161/HYPERTENSIONAHA.115.06011. 26195481 PMC4537368

[27] K. Wang , F. Liu , L. Y. Zhou , et al., “The Long Noncoding RNA CHRF Regulates Cardiac Hypertrophy by Targeting miR‐489,” Circulation Research 114, no. 9 (2014): 1377–1388, 10.1161/circresaha.114.302476. 24557880

[28] A. P. Owens, 3rd , D. L. Rateri , D. A. Howatt , et al., “MyD88 Deficiency Attenuates Angiotensin II‐Induced Abdominal Aortic Aneurysm Formation Independent of Signaling Through Toll‐Like Receptors 2 and 4,” Arteriosclerosis, Thrombosis, and Vascular Biology 31, no. 12 (2011): 2813–2819, 10.1161/ATVBAHA.111.238642. 21960563 PMC3220737

[29] T. Ha , F. Hua , Y. Li , et al., “Blockade of MyD88 Attenuates Cardiac Hypertrophy and Decreases Cardiac Myocyte Apoptosis in Pressure Overload‐Induced Cardiac Hypertrophy In Vivo,” American Journal of Physiology Heart and Circulatory Physiology 290, no. 3 (2006): H985–H994, 10.1152/ajpheart.00720.2005. 16199478

[30] A. L. Bayer , S. Smolgovsky , N. Ngwenyama , et al., “T‐Cell MyD88 Is a Novel Regulator of Cardiac Fibrosis Through Modulation of T‐Cell Activation,” Circulation Research 133, no. 5 (2023): 412–429, 10.1161/circresaha.123.323030. 37492941 PMC10529989

[31] P. Umbarkar , S. Tousif , A. Jaiswal , et al., “Fibroblast‐Specific MyD88‐Dependent Signaling Aggravates Inflammation and Cardiac Dysfunction in the MI Heart,” Biochimica et Biophysica Acta Molecular Basis of Disease 1871, no. 3 (2025): 167703, 10.1016/j.bbadis.2025.167703. 39894230 PMC12057582

[32] T. P. Mikolajczyk , P. Szczepaniak , F. Vidler , P. Maffia , G. J. Graham , and T. J. Guzik , “Role of Inflammatory Chemokines in Hypertension,” Pharmacology & Therapeutics 223 (2021): 107799, 10.1016/j.pharmthera.2020.107799. 33359600

[33] W. G. McMaster , A. Kirabo , M. S. Madhur , and D. G. Harrison , “Inflammation, Immunity, and Hypertensive End‐Organ Damage,” Circulation Research 116, no. 6 (2015): 1022–1033, 10.1161/circresaha.116.303697. 25767287 PMC4535695

[34] P. Wenzel , M. Knorr , S. Kossmann , et al., “Lysozyme M‐Positive Monocytes Mediate Angiotensin II‐Induced Arterial Hypertension and Vascular Dysfunction,” Circulation 124, no. 12 (2011): 1370–1381, 10.1161/circulationaha.111.034470. 21875910

[35] E. Girardin , G. E. Grau , J. M. Dayer , P. Roux‐Lombard , and P. H. Lambert , “Tumor Necrosis Factor and Interleukin‐1 in the Serum of Children With Severe Infectious Purpura,” New England Journal of Medicine 319, no. 7 (1988): 397–400, 10.1056/nejm198808183190703. 3135497

[36] A. Justin Rucker and S. D. Crowley , “The Role of Macrophages in Hypertension and Its Complications,” Pflugers Archiv: European Journal of Physiology 469, no. 3–4 (2017): 419–430, 10.1007/s00424-017-1950-x. 28251313 PMC5773253

[37] M. Ishibashi , K. Hiasa , Q. Zhao , et al., “Critical Role of Monocyte Chemoattractant Protein‐1 Receptor CCR2 on Monocytes in Hypertension‐Induced Vascular Inflammation and Remodeling,” Circulation Research 94, no. 9 (2004): 1203–1210, 10.1161/01.Res.0000126924.23467.A3. 15059935

[38] S. Nemska , M. Gassmann , M. L. Bang , N. Frossard , and R. Tavakoli , “Antagonizing the CX3CR1 Receptor Markedly Reduces Development of Cardiac Hypertrophy After Transverse Aortic Constriction in Mice,” Journal of Cardiovascular Pharmacology 78, no. 6 (2021): 792–801, 10.1097/fjc.0000000000001130. 34882111

[39] L. Ghita , J. Spanier , C. Chhatbar , et al., “MyD88 Signaling by Neurons Induces Chemokines That Recruit Protective Leukocytes to the Virus‐Infected CNS,” Science Immunology 6, no. 60 (2021): eabc9165, 10.1126/sciimmunol.abc9165. 34172587 PMC8717402

[40] M. Mühlbauer , E. Perez‐Chanona , and C. Jobin , “Epithelial Cell‐Specific MyD88 Signaling Mediates Ischemia/Reperfusion‐Induced Intestinal Injury Independent of Microbial Status,” Inflammatory Bowel Diseases 19, no. 13 (2013): 2857–2866, 10.1097/01.Mib.0000435445.96933.37. 24141713 PMC4039295

[41] K. Bandow , J. Kusuyama , M. Shamoto , K. Kakimoto , T. Ohnishi , and T. Matsuguchi , “LPS‐Induced Chemokine Expression in Both MyD88‐Dependent and ‐Independent Manners Is Regulated by Cot/Tpl2‐ERK Axis in Macrophages,” FEBS Letters 586, no. 10 (2012): 1540–1546, 10.1016/j.febslet.2012.04.018. 22673523

[42] H. S. Teixeira , J. Zhao , E. Kazmierski , D. F. Kinane , and M. R. Benakanakere , “TLR3‐Dependent Activation of TLR2 Endogenous Ligands via the MyD88 Signaling Pathway Augments the Innate Immune Response,” Cells 9, no. 8 (2020): 1910, 10.3390/cells9081910. 32824595 PMC7464415

[43] Y. Sun , M. Karmakar , S. Roy , et al., “TLR4 and TLR5 on Corneal Macrophages Regulate *Pseudomonas aeruginosa* Keratitis by Signaling Through MyD88‐Dependent and ‐Independent Pathways,” Journal of Immunology 185, no. 7 (2010): 4272–4283, 10.4049/jimmunol.1000874. PMC3392180 20826748

[44] N. Akhter , A. Hasan , S. Shenouda , et al., “TLR4/MyD88‐Mediated CCL2 Production by Lipopolysaccharide (Endotoxin): Implications for Metabolic Inflammation,” Journal of Diabetes and Metabolic Disorders 17, no. 1 (2018): 77–84, 10.1007/s40200-018-0341-y. 30288388 PMC6154519

