# Supplementary material for: Myeloid MyD88 Mediates Macrophage Infiltration and Activation in Ang II‐Induced Cardiac Hypertrophy
Source: J Cell Mol Med. 2025 Jul 19;29(14):e70733. doi: 10.1111/jcmm.70733 (PMC12274960; doi:10.1111/jcmm.70733)
Supplement: Supplementary file 1 — Data S1. [file JCMM-29-e70733-s001.docx]

***Supplementary Materials***

**Myeloid MyD88 mediates macrophage infiltration and activation in Ang II-induced cardiac hypertrophy**

**Supplementary Table S1.** Nucleotide sequences of qPCR primers.

| **Gene** | **Species** | **Description** | **Sequence (5’-3’)** |
| --- | --- | --- | --- |
| *Col1a1* | Mouse | Collagen type I α 1 chain | CTGTAACATGGAAACTGGGGAAA  CCATAGCTGAACTGAAAACCACC |
| *Myh7* | Mouse | Myosin heavy chain 7 | ACTGTCAACACTAAGAGGGTCA  TTGGATGATTTGATCTTCCAGGG |
| *Tgfb1* | Mouse | Transforming growth factor β1 | TGACGTCACTGGAGTTGTACGG  GGTTCATGTCATGGATGGTGC |
| *Il1b* | Mouse | Interleukin 1β | ACTCCTTAGTCCTCGGCCA  CCATCAGAGGCAAGGAGGAA |
| *Il6* | Mouse | Interleukin 6 | GAGGATACCACTCCCAACAGACC  AAGTGCATCATCGTTGTTCATACA |
| *Tnfa* | Mouse | Tumor necrosis factor α | CAGGGGCCACCACGCTCTTC  TTTGTGAGTGTGAGGGTCTGG |
| *Cxcl1* | Mouse | C-X-C motif ligand 1 | CTGGGATTCACCTCAAGAACATC  CAGGGTCAAGGCAAGCCTC |
| *Ccl2* | Mouse | C-C motif ligand 2 | TTAAAAACCTGGATCGGAACCAA  GCATTAGCTTCAGATTTACGGGT |
| *Actb* | Mouse | β actin | CCGTGAAAAGATGACCCAGA  TACGACCAGAGGCATACAG |


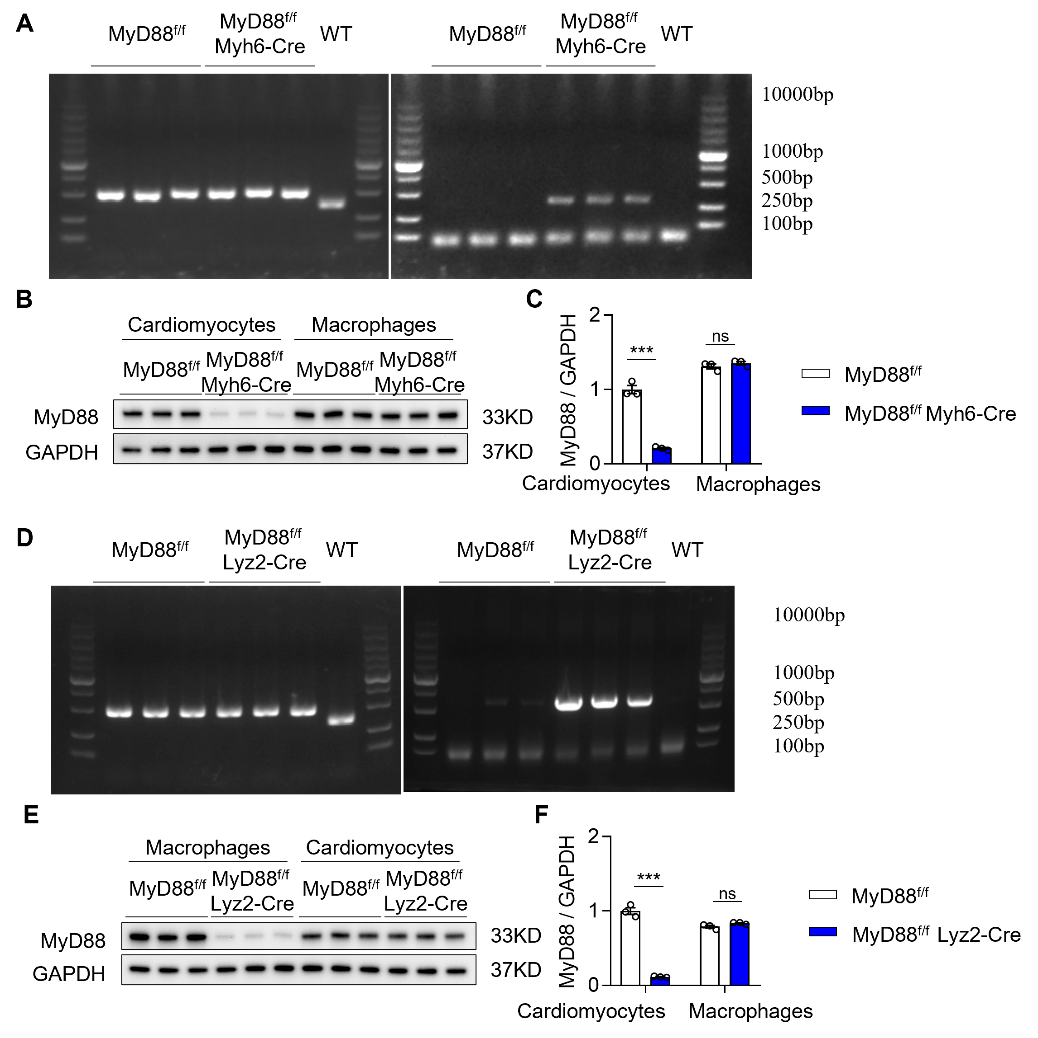


**Figure S1. The knockout efficiency and specificity of MyD88 *in vivo*.** (A) Genotyping of MyD88^f/f^ and MyD88^f/f^ Myh6-Cre mice as detected by tail-DNA extraction and DNA electrophoresis. (B) Representative Western blotting image of MyD88 in the primary neonatal murine cardiomyocytes and primary mouse peritoneal macrophages isolated from MyD88^f/f^ and MyD88^f/f^ Myh6-Cre mice. (C) Densitometric quantifications of the blots shown in (B). (D) Genotyping of MyD88^f/f^ and MyD88^f/f^ Lyz2-Cre mice as detected by tail-DNA extraction and DNA electrophoresis. (E) Representative Western blotting images of MyD88 in the primary neonatal murine cardiomyocytes and primary mouse peritoneal macrophages isolated from MyD88^f/f^ and MyD88^f/f^ Lyz2-Cre mice. (F) Densitometric quantifications of the blots shown in (E). Data are presented as mean ± SEM, *** *p* < 0.001, ns = not significant, compared to the MyD88^f/f^ mice.


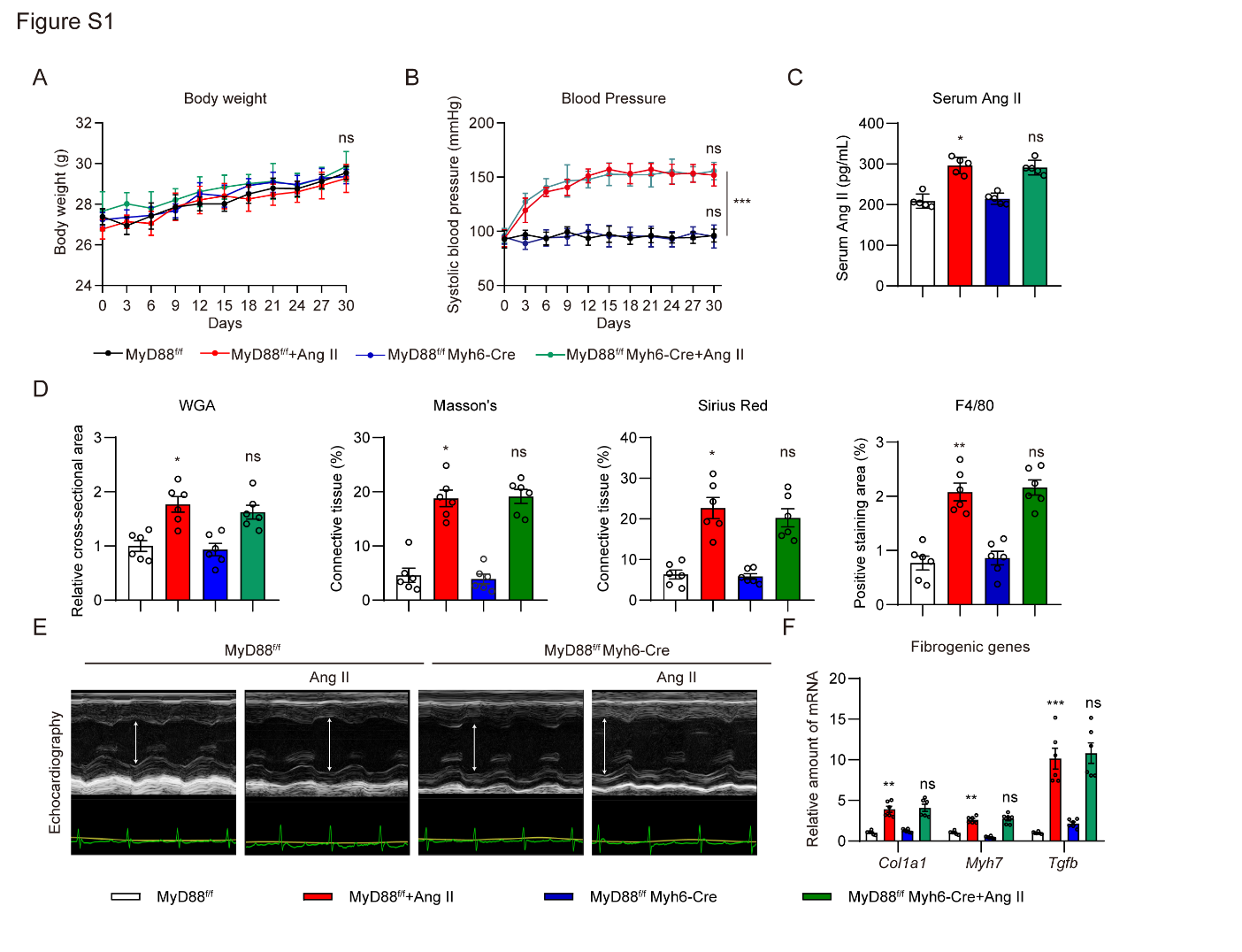


**Figure S2.** **Cardiomyocyte-specific knockout of MyD88 shows no significant improvements in Ang II-induced cardiac hypertrophy.** MyD88^f/f^ and MyD88^f/f^ Myh6-Cre mice were continuously challenged with Ang II (1 μg/kg/min) via micro-osmotic pumps for 4 weeks. Body weight (A) and blood pressure (B) of mice. (C) ELISA analysis of serum levels of angiotensin II. (D) Quantifications of histological staining results in Figure 2C-F. (E) Representative echocardiography of mice. (F) RT-qPCR analysis of fibrogenic genes in mice. Data are presented as mean ± SEM, n = 6; * *p* < 0.05, ** *p* < 0.01, *** *p* < 0.001, compared to the MyD88^f/f^ mice; ns = not significant, compared to the MyD88^f/f^ +Ang II mice.


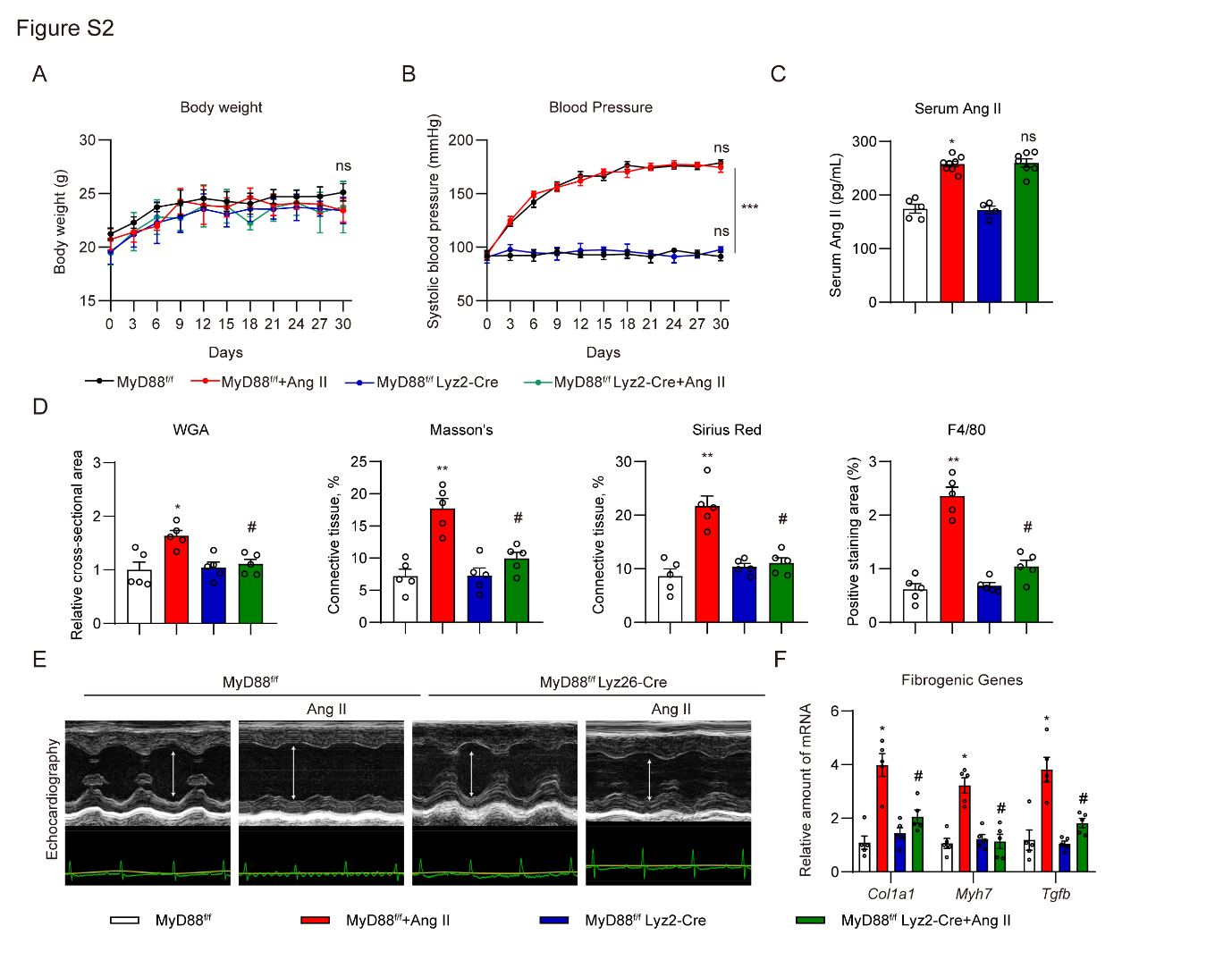


**Figure S3.** **Macrophage-specific knockout of MyD88 attenuates Ang II-induced cardiac hypertrophy.** MyD88^f/f^ and MyD88^f/f^ Lyz2-Cre mice were continuously challenged with Ang II (1 μg/kg/min) via micro-osmotic pumps for 4 weeks. Body weight (A) and blood pressure (B) of mice. (C) ELISA analysis of serum levels of angiotensin II. (D) Quantifications of histological staining results in Figure 3C-F. (E) Representative echocardiography of mice. (F) RT-qPCR analysis of fibrogenic genes in mice. Data are presented as mean ± SEM, n = 5; * *p* < 0.05, ** *p* < 0.01, ns = not significant, compared to the MyD88^f/f^ mice; # *p* < 0.05, compared to the MyD88^f/f^ +Ang II mice.


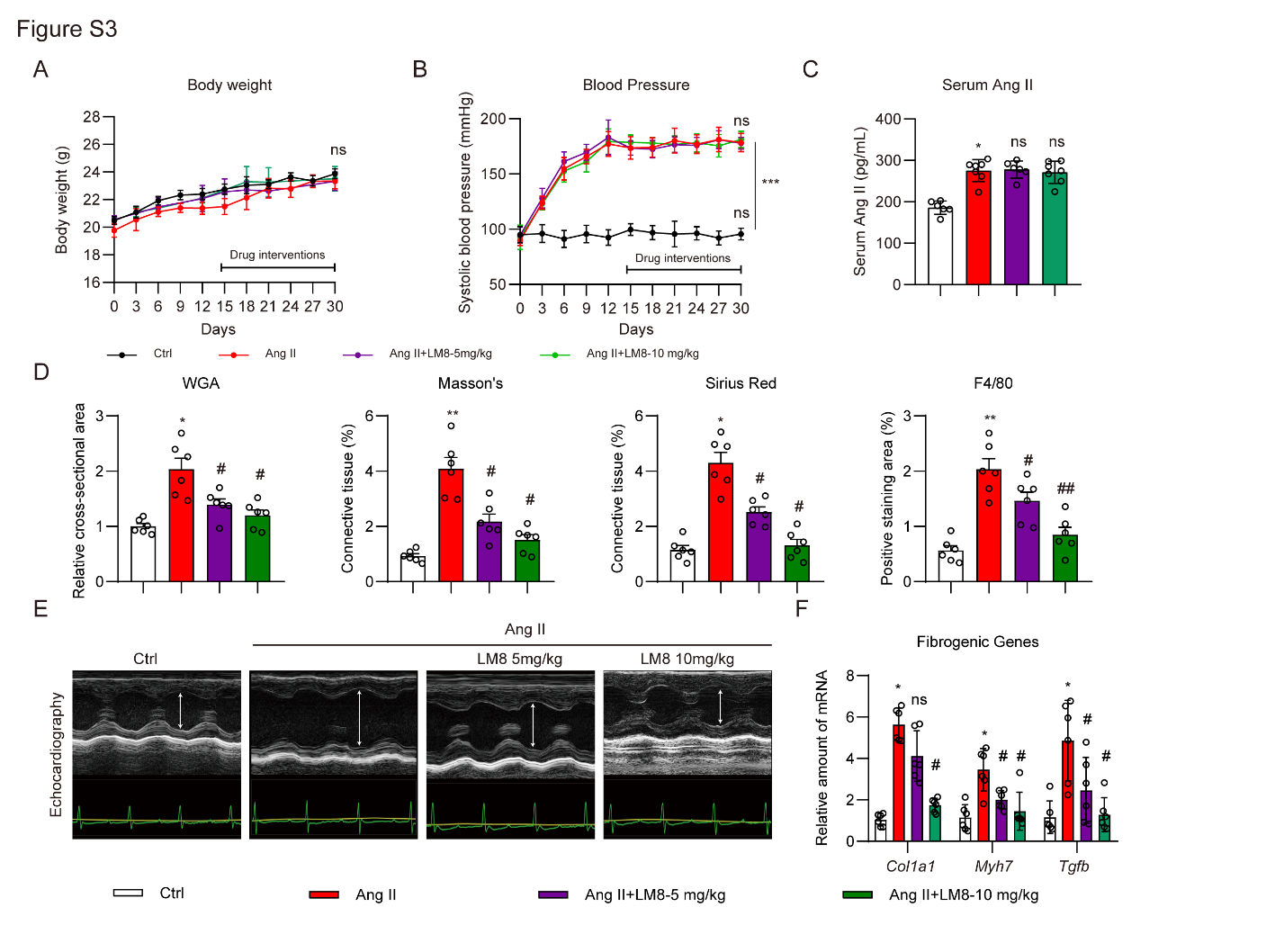


**Figure S4. Pharmacological inhibition of MyD88 ameliorates Ang II-induced cardiac hypertrophy.** C57BL/6 mice were continuously challenged with Ang II (1 μg/kg/min) via micro-osmotic pumps for 4 weeks to induce the hypertensive heart injury model. Two weeks after Ang II infusion, mice were daily treated with the MyD88 inhibitor (LM8, 5 or 10 mg/kg) or the vehicle control (DMSO) via intragastric injections. Body weight (A) and blood pressure (B) of mice. (C) ELISA analysis of serum levels of angiotensin II. (D) Quantifications of histological staining results in Figure 4C-F. (E) Representative echocardiograph of mice. (F) RT-qPCR analysis of fibrogenic genes in mice. Data are presented as mean ± SEM, n = 6; * *p* < 0.05, * *p* < 0.01, compared to the Ctrl mice; # *p* < 0.05, ## *p* < 0.01, ns = not significant, compared to the Ang II mice.


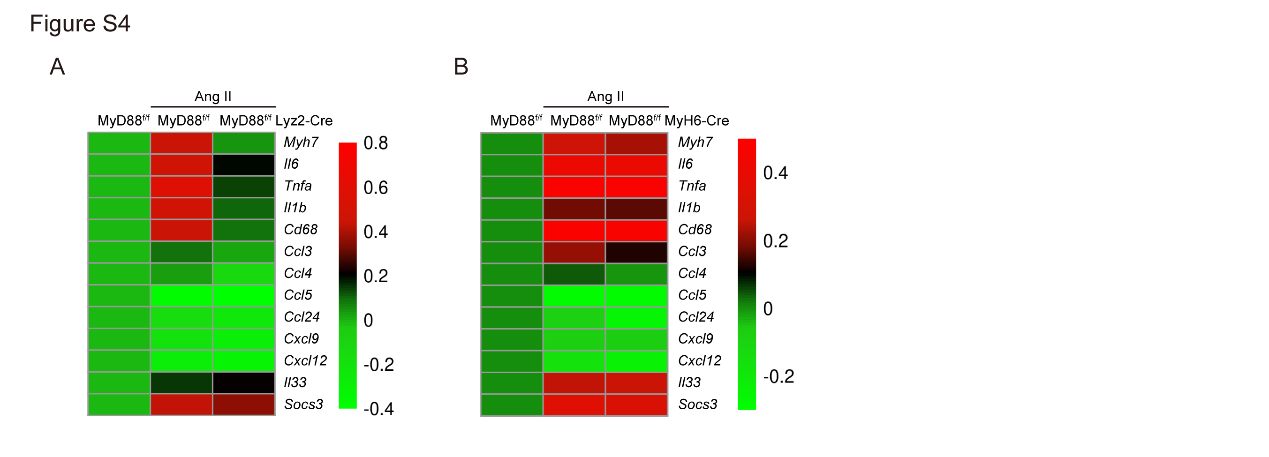


**Figure S5. Heat map of inflammatory chemokines and cytokines in heart tissue from MyD88 cardiomyocyte-specific knockout and MyD88 macrophage-specific knockout mice.** Heat map of inflammatory chemokines and cytokines in heart tissues from MyD88 macrophage-specific knock mice (A) and MyD88 cardiomyocyte-specific mice (B). cDNA from one group of mice were mixed and were used for RT-qPCR analysis. The results are shown as the heat map.


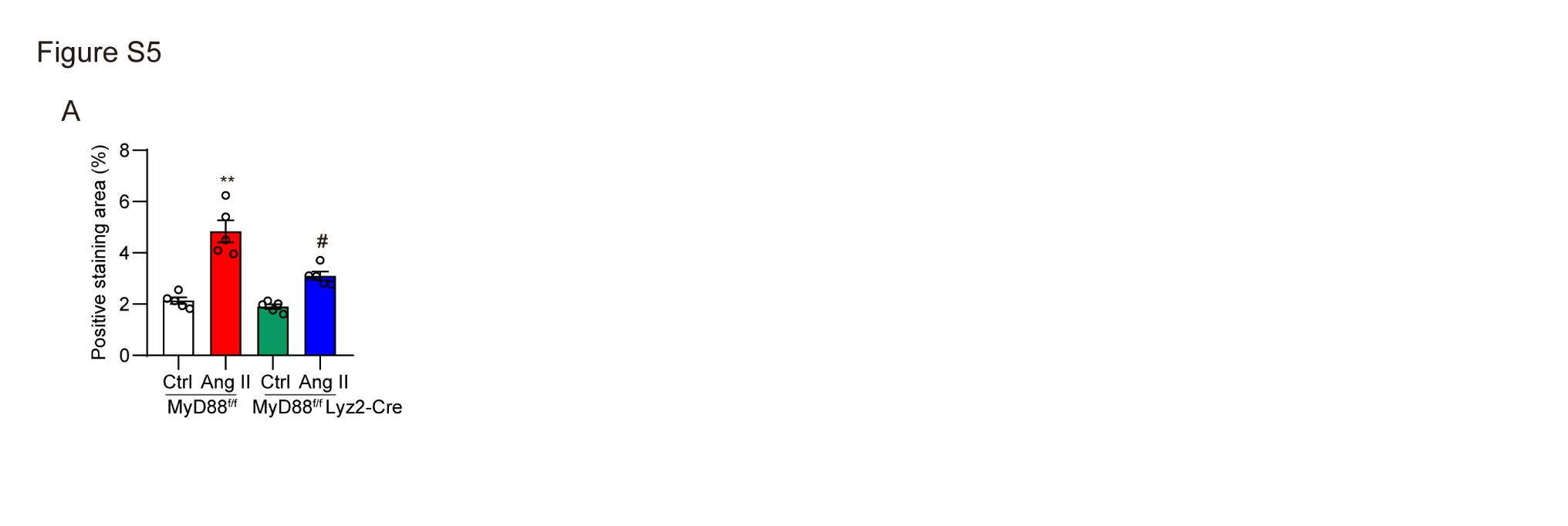


**Figure S6. MyD88 mediates Ang II-induced macrophage infiltration via regulating chemokine secretion.** (A) Quantifications of the data in Figure 5D. Data are presented as mean± SEM, ** *p* <0.01, compared to the Ctrl group; # *p* < 0.05, compared to the model group.
